# The SARS-CoV-2 neutralizing antibody response to SD1 and its evasion by BA.2.86

**DOI:** 10.1038/s41467-024-46982-6

**Published:** 2024-03-28

**Authors:** Daming Zhou, Piyada Supasa, Chang Liu, Aiste Dijokaite-Guraliuc, Helen M. E. Duyvesteyn, Muneeswaran Selvaraj, Alexander J. Mentzer, Raksha Das, Wanwisa Dejnirattisai, Nigel Temperton, Paul Klenerman, Susanna J. Dunachie, Elizabeth E. Fry, Juthathip Mongkolsapaya, Jingshan Ren, David I. Stuart, Gavin R. Screaton

**Affiliations:** 1 Chinese Academy of Medical Science (CAMS) Oxford Institute (COI), University of Oxford, Oxford, UK; 2 Division of Structural Biology, Nuffield Department of Medicine, University of Oxford, Centre for Human Genetics, Oxford, UK; 3 Centre for Human Genetics, Nuffield Department of Medicine, University of Oxford, Oxford, UK; 4 NIHR Oxford Biomedical Research Centre, Oxford University Hospitals NHS Foundation Trust, Oxford, UK; 5 Division of Emerging Infectious Disease, Research Department, Faculty of Medicine Siriraj Hospital, Mahidol University, Bangkok-Noi, Bangkok, 10700 Thailand; 6 Viral Pseudotype Unit, Medway School of Pharmacy, University of Kent and Greenwich Chatham Maritime, Kent, ME4 4TB UK; 7 Peter Medawar Building for Pathogen Research, University of Oxford, Oxford, UK; 8 Translational Gastroenterology Unit, Nuffield Department of Medicine, University of Oxford, Oxford, UK; 9 NDM Centre For Global Health Research, Nuffield Department of Medicine, University of Oxford, Oxford, UK; 10 Mahidol-Oxford Tropical Medicine Research Unit, Bangkok, Thailand; 11 Diamond Light Source Ltd, Harwell Science & Innovation Campus, Didcot, UK; 12 Present Address: College of Life Sciences, Zhejiang University, Hangzhou, 310058 China

**Keywords:** SARS-CoV-2, Viral infection, Biophysics, Viral infection

## Abstract

Under pressure from neutralising antibodies induced by vaccination or infection the SARS-CoV-2 spike gene has become a hotspot for evolutionary change, leading to the failure of all mAbs developed for clinical use. Most potent antibodies bind to the receptor binding domain which has become heavily mutated. Here we study responses to a conserved epitope in sub-domain-1 (SD1) of spike which have become more prominent because of mutational escape from antibodies directed to the receptor binding domain. Some SD1 reactive mAbs show potent and broad neutralization of SARS-CoV-2 variants. We structurally map the dominant SD1 epitope and provide a mechanism of action by blocking interaction with ACE2. Mutations in SD1 have not been sustained to date, but one, E554K, leads to escape from mAbs. This mutation has now emerged in several sublineages including BA.2.86, reflecting selection pressure on the virus exerted by the increasing prominence of the anti-SD1 response.

## Abstract (web summary)

Due to the focus of vaccination on the SARS CoV-2 spike protein, spike has been associated with high levels of viral mutation and subsequent immune escape. Here the authors study a conserved epitope in SARS CoV-2 sub-domain-1 and characterise the neutralising antibody response and evasion in contemporary SARS COV-2 viral strains.

## Introduction

Since the emergence of SARS-CoV-2 in late 2019, there have been roughly 772 million documented infections and 7 million deaths^1^, however, it is believed that these numbers are underestimates and that the majority of the human population has now been vaccinated against and/or infected with SARS-CoV-2, often on multiple occasions. The resultant widespread herd immunity has exerted very strong selective pressure on SARS-CoV-2 to evade neutralizing antibody responses in order to re-infect previously exposed individuals and maintain productive infection cycles in the human population^2–5^.

**Fig. 1:**
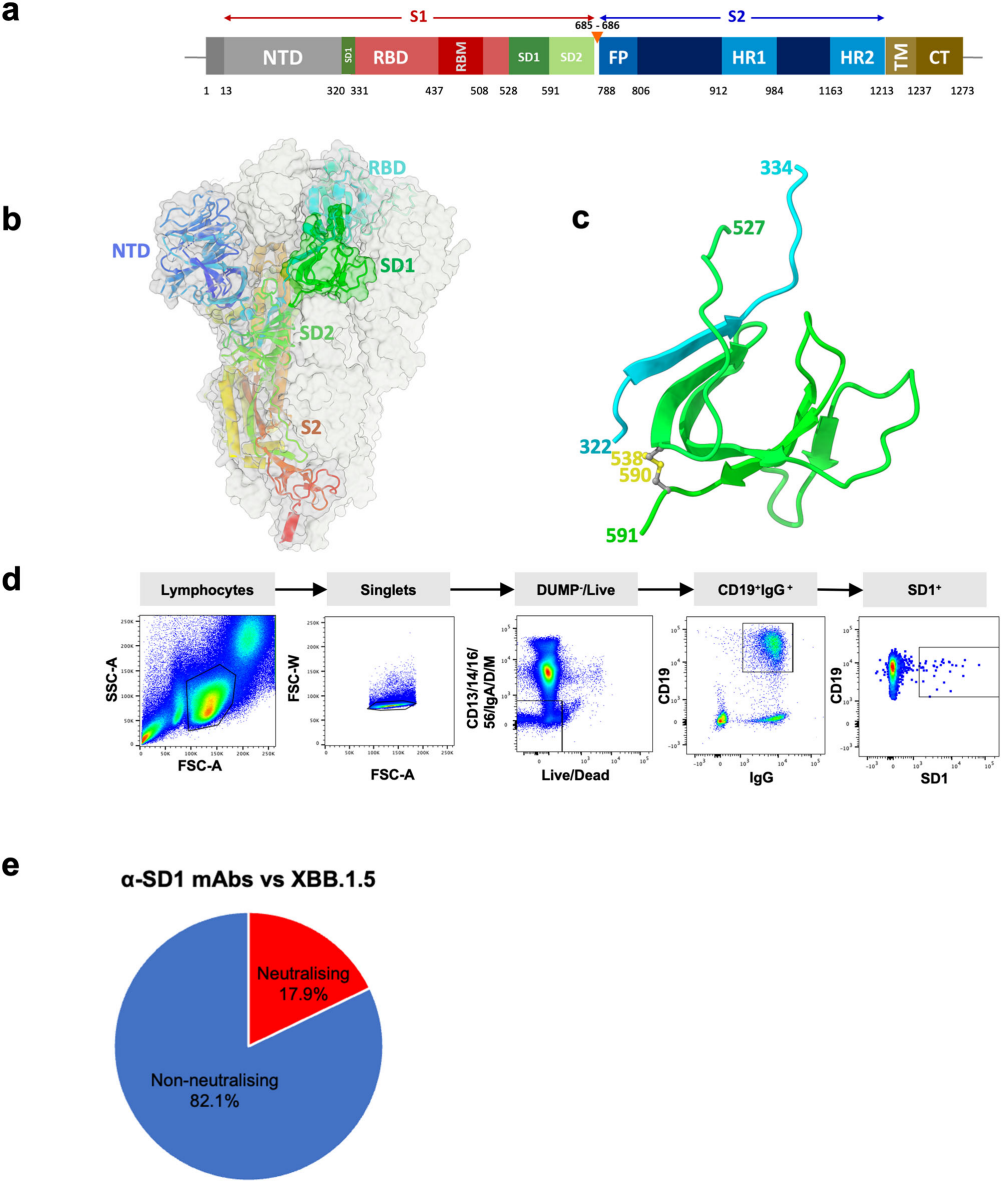
Structure of SD1 and generation of anti-SD1 mAbs. **a** Linear map of S marking S1 and S2 and showing elements of SD1 flanking RBD at both ends. **b** Structure of the S trimer showing positions of SD1, SD2, NTD, and RBD in S1. **c** Close up of structure of SD1 showing the N-terminal loop 322- 334 in cyan and the C-terminal fragment 527–591 in light green, the position of an intrachain disulfide bond is shown in yellow. **d** FACS sorting strategy used to isolate SD1 reactive memory B cells. **e** Neutralization potential of anti-SD1 mAbs. Non-neutralizing mAb (IC50 >10 μg/ml) do not achieve >50% neutralizing activity against Victoria and XBB.1.5 live virus at a concentration of 10 μg/ml.

The spike protein (S) is the site for binding of neutralizing antibodies and analysis of panels of human monoclonal antibodies (mAbs) generated from infected volunteers has shown that the majority of the most potent mAbs bind to the receptor binding domain (RBD) in subunit 1 (S1) of S (Fig. 1a–c)^6–8^. Most potent anti-RBD mAbs bind on or in close proximity to the receptor binding motif ^9–11^, blocking the interaction of RBD with the cellular SARS-CoV-2 receptor, angiotensin-converting enzyme 2 (ACE2)^12^, although a few bind elsewhere and may function to destabilize the S trimer^13–15^. A number of potent mAbs bind to a so-called supersite in the N-terminal domain (NTD) of S1, although their mechanism of neutralization is poorly understood^16,17^ and some bind at the interface of the NTD and SD1 locking the RBDs down to prevent ACE2 interaction^18^.

The binding sites for potently neutralizing mAbs have been hotspots for mutations within the NTD and RBD, leading to large falls in the neutralization titers in serum obtained from both vaccinees and naturally infected cases^19–21^. Mutational change in the RBD has also led to the loss of activity of mAbs developed for clinical use^22^ leading to a search for potent and broadly reacting antibodies binding to more conserved or stable epitopes among SARS-CoV-2 variants.

In this study we report the generation of a panel of mAbs that have arisen from infection or vaccination, binding outside the NTD and RBD in sub-domain-1 (SD1), a highly conserved domain adjacent to the RBD. Some of these anti-SD1 mAbs show broad and potent neutralization of many SARS-CoV-2 variants. We selected three potent anti-SD1 mAbs for further study and determined their structures in complex with the S trimer. We suggest they function by blocking the interaction of S with ACE2. Depletion of the SD1 reactive antibodies from serum shows that the relative contribution of the anti-SD1 response to overall neutralization titers has increased when the neutralization of contemporary viruses is compared to early pandemic viruses. The increased pressure exerted by the anti-SD1 response likely explains the emergence of mutation E to K at residue 554, which abrogates the binding of all our potent anti-SD1 antibodies, in several very recently reported SARS-CoV-2 sequences, including BA.2.86^23^.

## Results

### Generation of SD1 reactive mAbs

We initially isolated an SD1 reactive mAb, SD1-1, from a vaccinated donor who had suffered a vaccine breakthrough BA.5 infection. Memory cells were single cells sorted from B cells stained with BA.4S trimers. Following a degenerate PCR reaction, heavy and light chains were assembled into an expression vector, and products were expressed by transient transfection. Supernatants were tested for binding to full-length BA.4S, BA.4 RBD, and BA.4 NTD. SD1-1 reacted to full-length S but not RBD or NTD, and its functional and structural characterization will be described below.

SD1 is a small domain present in the S1 subunit of spike (Fig. 1a–c), formed from residues 320–331 lying N-terminal to the RBD and 528–591, C-terminal to the RBD. To produce a recombinant SD1 domain, we connected residue 331 with 528 using a nine-residue long gly-gly-ser linker. SD1 was expressed in 293T cells and purified using a C-terminal double strep tag. Purified SD1 was tested for reactivity to mAb SD1-1 by ELISA and then used to stain and sort memory B cells from vaccinated donors suffering from breakthrough SARS-CoV-2 infections with BA.4/5 or later variants (Fig. 1d).

Antibody supernatants were tested for reactivity to SD1 and XBB.1.5S by ELISA, leading to the identification of 26 SD1 reactive mAbs from 348 sorted B cells. Supernatants from the initial transfection of the products of Gibson assembly were also tested in live virus neutralization assays against Victoria^24^ (an early pandemic strain) and XBB.1.5. From this, we found that the majority of SD1 reactive mAbs showed little or no neutralization of SARS-CoV-2, IC50 >10 μg/mL against Victoria and XBB.1.5 in live virus neutralization assays. (Fig. 1e). Four anti-SD1-mAbs were isolated that showed potent neutralization (IC50 <100 ng/ml): SD1-1 to SD1-4. SD1-1, SD1-3, and SD1-4 are from two BA.4/5 infected vaccine breakthrough samples, while SD1-2 is from an XBB.1.5 vaccine breakthrough sample. SD1-3 and SD1-4 (both isolated from the same donor) were highly related (Supplementary Table 4), showing only a single aa difference in the light chain, outside the CDRs, so we focussed our subsequent studies on SD1-1, SD1-2, and SD1-3.

### Broad neutralization by SD1 mAbs

**Fig. 2:**
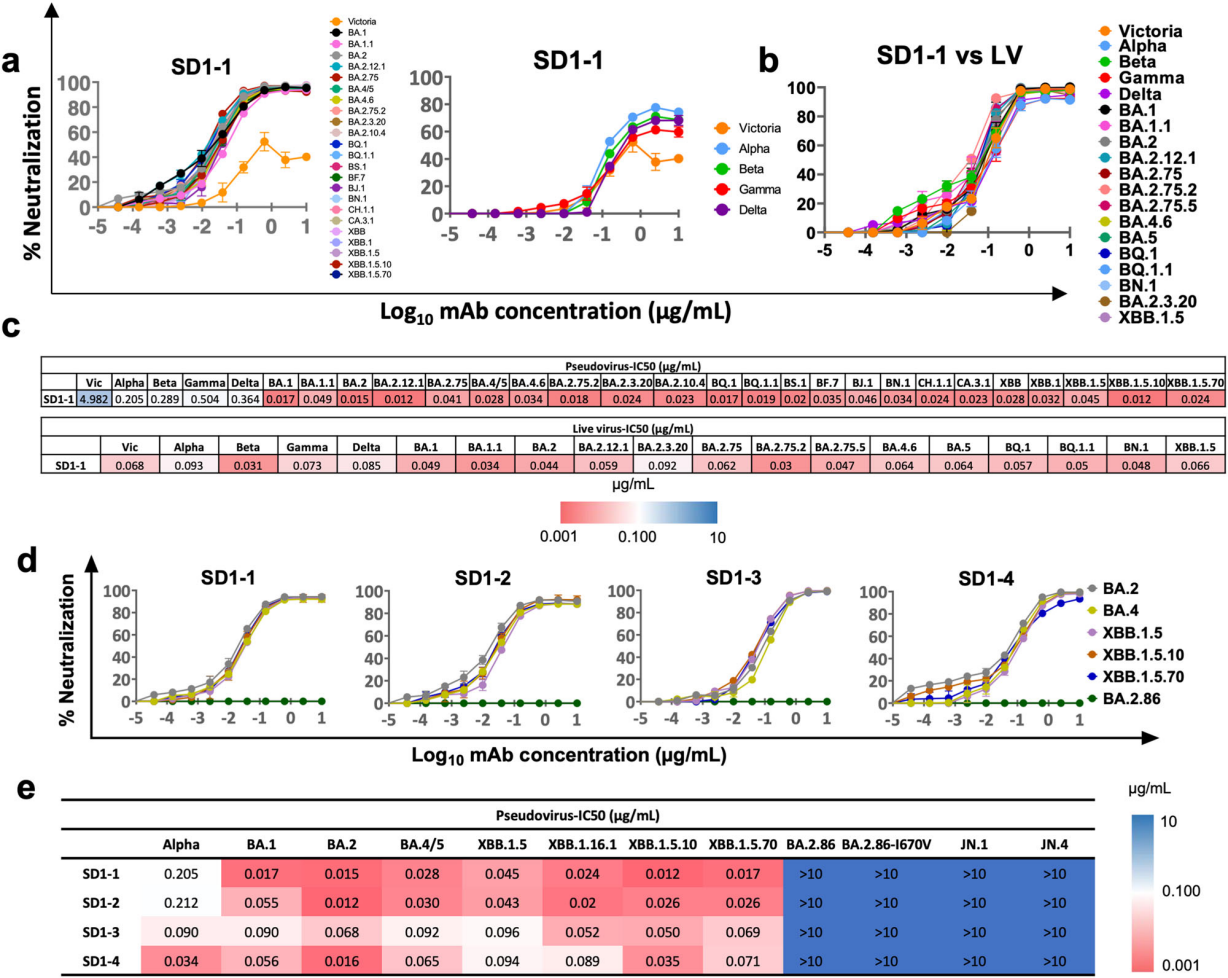
Broad neutralization of SARS-CoV-2 variants by anti-SD1 mAbs. **a** Titration curves for neutralization of a variety of pseudoviral constructs by SD1-1. **b** Live virus neutralization curves using SD1-1 with the indicated viruses. **c** Heatmap of IC50 values for the indicated pseudoviruses and live viruses. **d**, **e** Neutralization curves and IC50 values against the indicated pseudovirus variants. *n* = 2 independent experiments with duplicates. Data are presented as mean values ± SEM. Source data are provided as a Source Data file.

First, we tested the activity of SD1-1 in pseudovirus neutralization assays^25^ against a large panel of variants seen throughout the pandemic^26^ with particular emphasis on Omicron sublineages (Fig. 2). The sequence of SD1 in all these variants is identical (apart from A570D in Alpha and the T547K mutation observed in BA.1 and BA.1.1). SD1-1 showed activity against all the Omicron sublineages with NT50 titers ranging from 12 to 45 ng/ml (Fig. 2c). Victoria, Alpha, Beta, Gamma, and Delta all showed a plateau of neutralization below 80% with SD1-1 (Fig. 2a). To determine whether this was an artifact of the pseudovirus assay we performed neutralization assays using live viruses, where neutralization plateaued above 90% (Fig. 2b). The reason for the reduced neutralization of early SARS-CoV-2 variants Victoria, Alpha, Beta, Gamma and Delta by anti-SD1 mAb compared to wild-type viruses remains obscure, but we have previously confirmed that these early SARS-CoV-2 variants are well neutralized by a number of well characterized mAbs, although we have previously noted that an anti-NTD mAb, 159 was unable to neutralize Victoria pseudoviral particles but showed potent neutralization against live virus^20^.

Next, we tested neutralization by SD1-1 using a smaller set of pseudoviruses representing more contemporary Omicron sublineages, again showing potent neutralizing activity with IC50 <100 ng/ml (Fig. 2d, e). Finally, to test whether neutralization mediated via the SD1 epitope required bivalent binding of mAb, we tested neutralization using Fab of the anti-SD1 mAb. This demonstrated that all SD1 Fab could neutralize SARS-CoV-2 variants: Victoria, Alpha, BA.1, and BA.5, albeit neutralization titers were reduced 18.5, 2.6, and 11.8-fold for SD1-1, SD1-2 and SD1-3 Fabs respectively compared to IgG1 on Victoria virus (Supplementary Fig. 4c).

### ACE2 receptor blocking activity of anti-SD1 mAbs

**Fig. 3:**
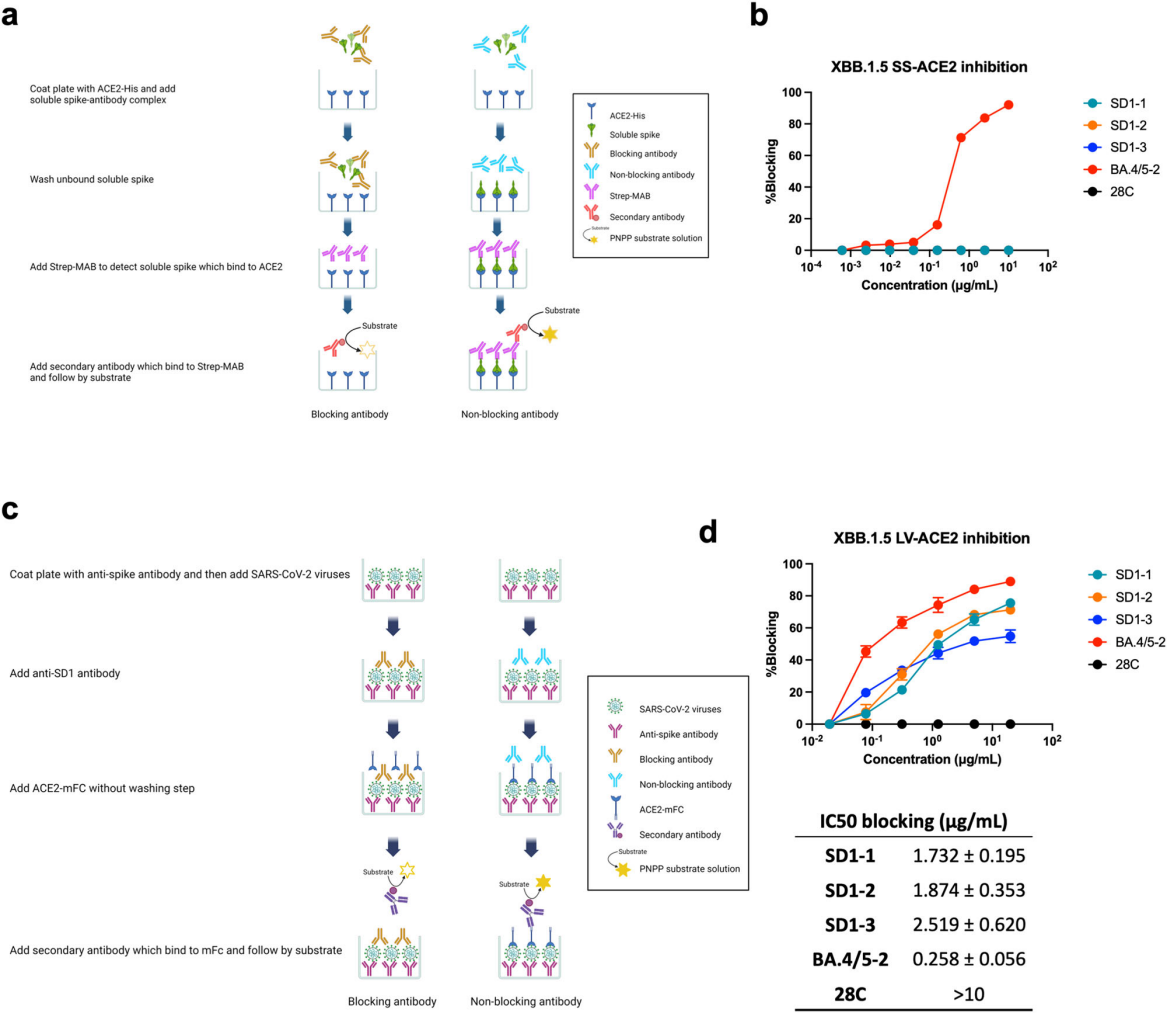
ACE2 blocking by anti-SD1 mAbs. **a** Schematic for the ACE2 blocking assay using recombinant S (created with BioRender.com). **b** Blocking of ACE2 binding to XBB.1.5S by anti-RBD mAb BA.4/5-2 but not by anti-SD1 mAbs, 28C is a negative control anti-influenza mAb. **c** Schematic for the virion ACE2 blocking assay (created with BioRender.com). **d** ACE2 binding to XBB.1.5S protein is blocked by anti-SD1 mAbs. *n* = 2 independent experiments with duplicates. Data are presented as mean values ± SEM. Source data are provided as a Source Data file.

The majority of potent SARS-CoV-2 mAbs function to block the interaction of S with the ACE2 receptor^6,8,18^, although some, such as S309^27^, which binds distant from the ACE2 interaction surface or those binding to the supersite in the NTD^28^ show potent neutralizing activity but do not antagonize ACE2 interaction. We tested the ability of the anti-SD1 mAbs and Fabs to antagonize ACE2 binding to XBB.1.5S using an ELISA-based assay (Fig. 3a) using mAb BA.4/5-2, which binds the ACE2 interacting surface of Wuhan S and can bind simultaneously with SD1-1 (Supplementary Fig. 1a) as a positive control and mAb 2-8C^29^, an anti-flu mAb, as a negative control (Fig. 3b). Using this assay format, in which ACE2 is directly attached to the ELISA plate, we found that the SD1 mAbs (IgG1 or Fab) failed to block the binding of soluble spike to ACE2.

We repeated this assay in a different format to determine whether anti-SD1 mAb could still bind to S already loaded with ACE2. In this assay S was bound onto the ELISA plates, incubated with a titration of ACE2-mouse Fc, and then mAb were added, and binding was revealed using anti-human IgG-AP (Supplementary Fig. 4a), and in a separate lane, the binding of ACE2-mouse Fc was revealed by anti-mouse IgG-AP (Supplementary Fig. 4b). Binding of anti-SD1 mAb was unaffected by the ACE2 titration, however, binding of mAb222, an ACE2 blocking mAb that binds to the RBD was also unaffected^6^. We went on to determine whether mAb222 was able to bind by displacing ACE2 from S by determining the residual presence of ACE2-mouse Fc bound to S, which showed that mAb222 could displace ACE2 but that anti-SD1 mAb could not (Supplementary Fig. 4b) leading us to conclude that anti-SD1 mAb binding to S was independent of ACE2 when binding to recombinant soluble spike complexes.

We wondered whether anti-SD1 mAbs may function differently on intact virion particles. To test this, we devised an assay using whole viral particles; XBB.1.5 virions were captured onto ELISA plates coated with mAb166^6^ reactive to SARS-CoV-2 S2. Plates were then incubated with SD1 mAbs or control mAbs followed by addition of ACE2-mouse Fc, the binding of which was revealed by anti-mouse Fc-AP (Fig. 3c). Using this whole virion assay, anti-SD1 mAbs (IgG1 and Fab) showed ACE2 blocking activity, with the two most potent neutralizers SD1-1 and SD1-2 showing the most potent activity (Fig. 3d and Supplementary Fig. 4d).

### Structures of spike and anti-SD1 Fab complexes

**Fig. 4:**
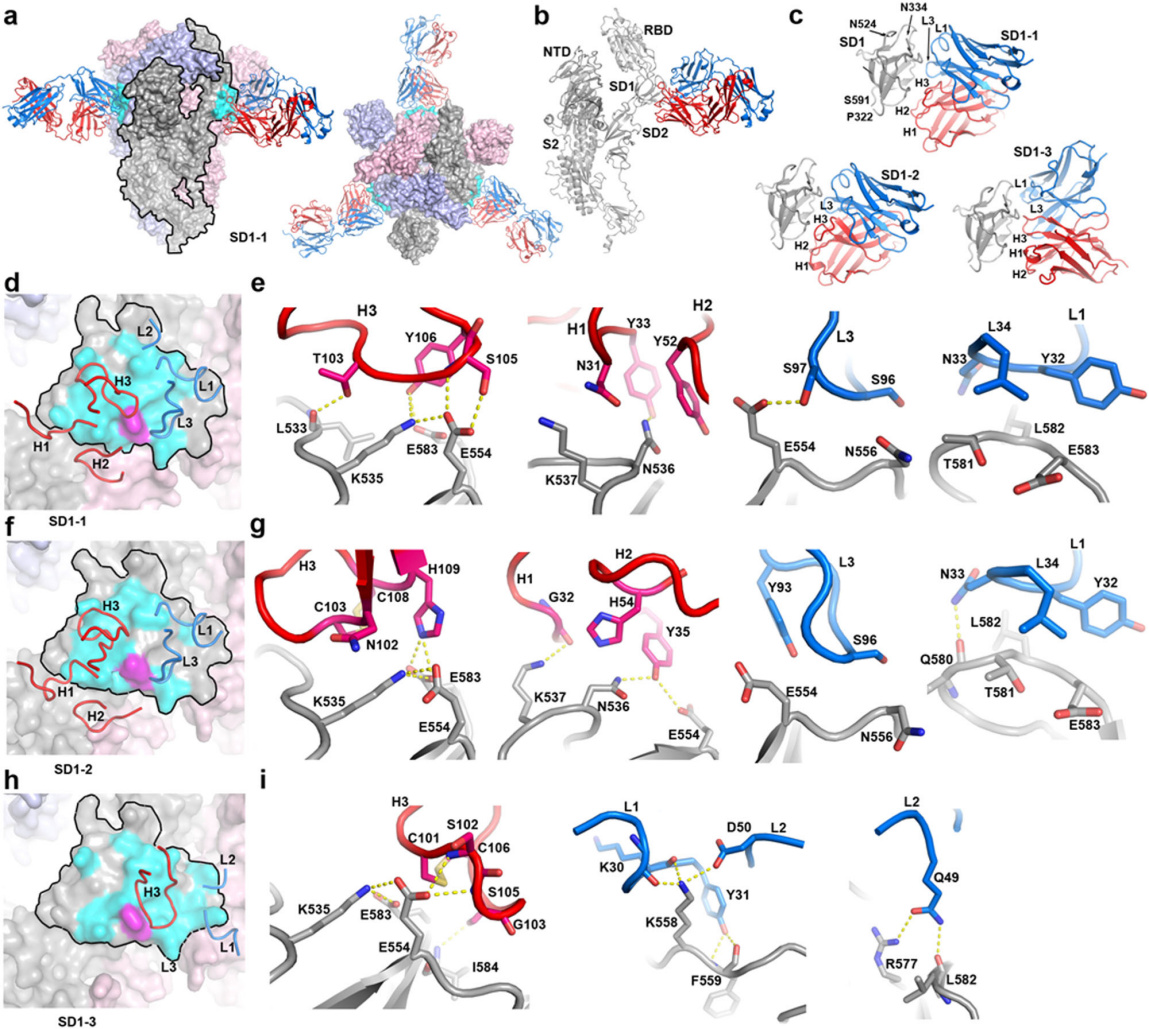
Structures of SD1 Fabs bound with the SARS-CoV-2 spike. **a** Side (left) and top (right) views of BA.4 spike and SD1-1 Fab complex. The trimeric spike is shown as a surface representation with chains colored in gray, salmon, and pale blue. The bound Fabs are shown as ribbons colored in red for HC and blue for LC, and its footprint on the spike in cyan. One chain of the spike is outlined by black lines. **b** Ribbon representation of the outlined chain of the spike (gray) in (**a**) and its bound Fab. **c** Binding mode comparison of SD1-1 (top) with SD1-2 (lower left) and SD1-3 (lower right) by aligning the SD1 domain. The latter two Fabs are complexed with BA.2.12.1 spike (full structures are not shown for simplicity). **d**, **e** Binding position and orientation of the CDRs relative to SD1 domain (outlined) and details of interactions with SD1 for SD1-1, **f**, **g** for SD1-2, **h**, **i** for SD1-3. In **d**, **f**, **h**, CDRs which have direct contact with the spike (≤4.0 Å) are shown, and E554 that is mutated to a lysine in BA.2.86 is highlighted in magenta. Protein main chains are drawn as ribbons and coils, and side chains as sticks with Fab HC in red and LC in blue, SD1 in gray. Hydrogen bonds are shown as yellow broken sticks.

To determine the binding modes and details of the interaction of these potent neutralizing mAbs with S, we determined high-resolution cryo-EM structures of complexes of BA.4/5 spike with SD1-1 Fab and BA.2.12.1 spike with SD1-2 and SD1-3 Fabs (Supplementary Table 1 and Fig. 4). As expected, all three Fabs bind the SD1 domain with a 1:3 (spike:Fab) stoichiometry. In all cases, the spike is in a three-RBD down conformation and is essentially threefold symmetric, with the Fabs contacting residues only from the SD1 domain (Fig. 4a, b). A triad of three charged residues, K535, E554, and E583, forms the key attachment point for all these anti-SD1 mAbs.

SD1-1 is an IGHV4-59 antibody (Supplementary Table 2) and uses all the CDRs for binding with SD1 (Fig. 4d). Detailed analysis of the interaction of SD1-1 Fab with the SD1 domain is presented in Fig. 4d, e. The interaction of the CDR-H3 loop is particularly extensive, with six potential hydrogen bonds formed and interaction made with three portions of the SD1 domain: the loop from N532 to K535, residue E554 and residue E583, all other CDR loops interact to a lesser extent, with CDR-L2 making the least interactions (Fig. 4e). The total interaction area is 690 Å^2^. In addition, the glycan attached to residue N333 contacts the antibody, but makes only weak interactions.

SD1-2, an IGHV4-61 antibody (Supplementary Table 2), binds in a similar position and orientation to SD1-1 (Fig. 4f), but shares only 72% heavy chain Vh sequence identity. In addition, the CDR-H1 and H3 of SD1-2 are two residues and one residue longer, respectively, than those of SD1-1. SD1-2 makes a footprint of 660 Å^2^ on SD1, slightly smaller than SD1-1. In the unliganded SD1 of the BA.2.12.1 spike (PDB: 8CIM), K535 makes a salt bridge to E583 of the 535, 554, 583 triad. Upon binding of SD1-2, K535 forms salt-bridges to both E554 and E583, both of which, in turn, salt-bridge to H109 of CDR-H3 (Fig. 4g). The disulfide linking the 6th and 11th positions of CDR-H3 makes hydrophobic contacts with residue L533 of CD1. CDR-L2 is not involved in interactions, and other CDRs make SD1 contacts similar to the corresponding mAb SD1-1 CDRs (Fig. 4e, g).

Fab SD1-3 (IGHV3-23, Supplementary Table 2) binds SD1 using only three CDRs, H3, L1, and L2, and makes a smaller footprint of 560 Å^2^ (Fig. 4h). Its CDR-H3 overlaps partially with those of SD1-1 and SD1-2, making hydrogen bonds from the amino groups of S102 and G103 to E554 of the SD1 triad (Fig. 4i). However, Fab SD1-3 is rotated clockwise by about 60° relative to SD1-1 (Fig. 4c). Interestingly, the CDR-H3 of SD1-3 also contains a disulfide linking the fifth and tenth positions, which makes direct contacts with the triad. CDRs L1 and L2 interact with residues K557-F559, R577, and L582.

Several anti-SD1 mAbs have been reported previously, and structures are available for three, S3H3, P008-60, and SD1.040, all of which are much less potent than those described here^30–33^. S3H3 binds a similar region to mAb SD1-1 but is rotated on the antigen by about 90°, although the interaction patch on SD1 for the heavy chain CDR3 (H3) is similar for both antibodies (Supplementary Fig. 2a, b). In contrast, P008-60 binds towards the top of the SD1 domain (although an extended CDR-H3 hairpin points downwards towards the region where the CDR-H3 loops of the other two SD1 antibodies interact) (Supplementary Fig. 2c). P008-60 binding sterically interferes with the NTD of the adjacent subunit in the trimer, causing disruption of the spike. SD1.040 binds in an intermediate position, higher on SD1-1 (Supplementary Fig. 2d)^32^. SD1.040 also destabilizes the prefusion trimer, and, since SD1.040 did not appear to compete with ACE2 binding, this destabilization may be the mechanism of neutralization. In contrast, S3H3 and all our anti-SD1 mAbs project outwards perpendicular to the threefold axis of the spike, avoiding clashes with adjacent domains in the trimer, and presumably neutralization is via the blocking of ACE2 attachment to the virion, with stabilization of the three-down prefusion spike also potentially contributing^25^.

### Quantifying the role of the anti-SD1 response in polyclonal serum

**Fig. 5:**
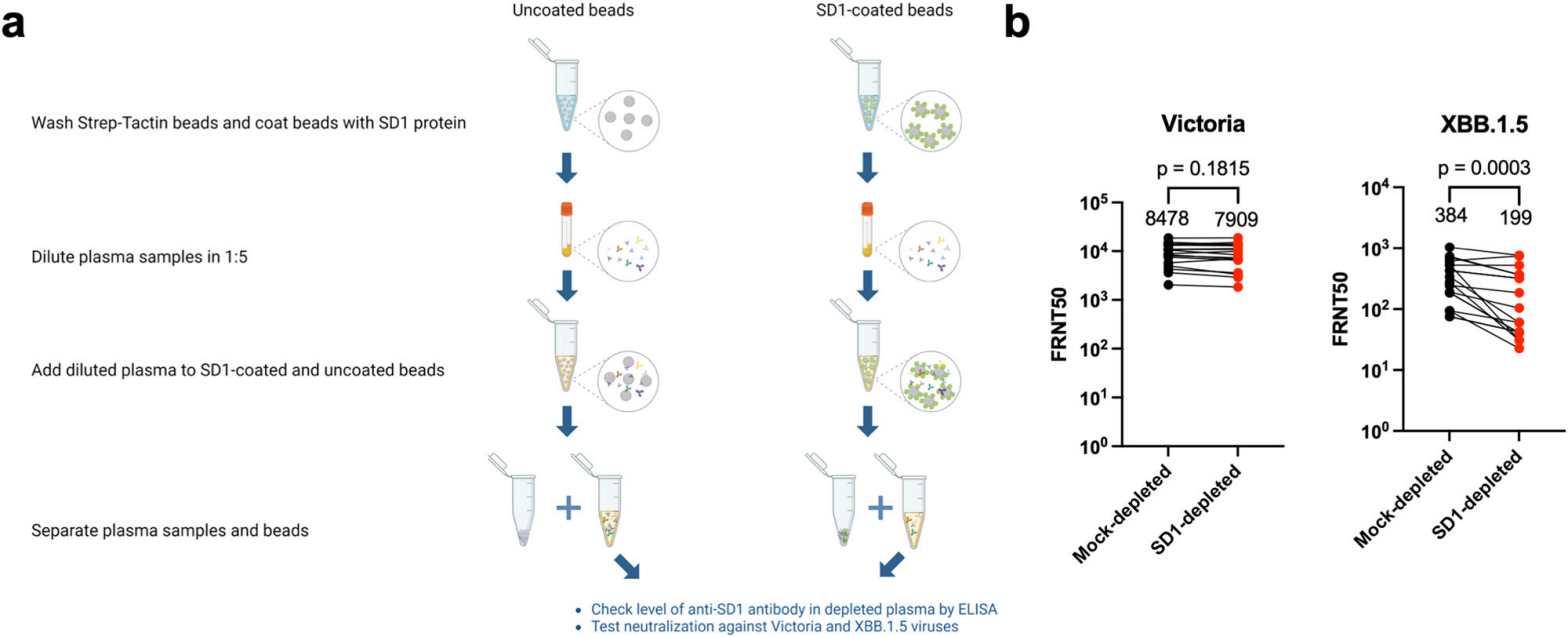
Depletion of SD1 reactive antibody. **a** Schematic showing SD1 depletion (created with BioRender.com)**. b** Live virus neutralization assays were performed against an early pandemic strain Victoria and later strain XBB.1.5, mock depleted, and SD1 depleted samples are compared. Geometric means are shown in each column and Wilcoxon matched-pairs signed-rank test was used for the analysis, and two-tailed *P* values were calculated. Source data are provided as a Source Data file.

To determine whether anti-SD1 antibodies play a significant role in the polyclonal response to SARS-CoV-2, we depleted polyclonal sera of anti-SD1 activity using recombinant SD1 protein (Fig. 5a). Sera from 18 volunteers was obtained between March 2022 and February 2023, following vaccine breakthrough infection with more recent omicron sublineages (Supplementary Table 3). Serum samples were incubated with beads coated with recombinant SD1 protein, or with mock beads without coating. Following the removal of beads, depletion of SD1 reactivity was confirmed by ELISA against SD1-1 mAb.

SD1 and mock depleted sera were then used in live virus neutralization assays against the early pandemic strain Victoria and the Omicron sub-lineage XBB.1.5. Depletion of SD1 showed a non-significant 1.07-fold fall (*p* = 0.1815) in neutralization titers to Victoria, but a highly significant 1.9-fold fall in titers to XBB.1.5 (*p* = 0.0003) (Fig. 5b). Titers to Victoria were 22-fold higher than titers to XBB.1.5 with the former likely representing the response induced by vaccination. As much of the neutralization activity to Victoria is knocked out by mutations in XBB.1.5, the fraction of anti-SD1 activity in serum is likely to rise, and it may also be that boosting of pre-existing anti-SD-1 responses by more contemporary viruses such as BA.4/5 leads to further expansion of the anti-SD1 response.

### Mutational escape from anti-SD1 mAbs

**Fig. 6:**
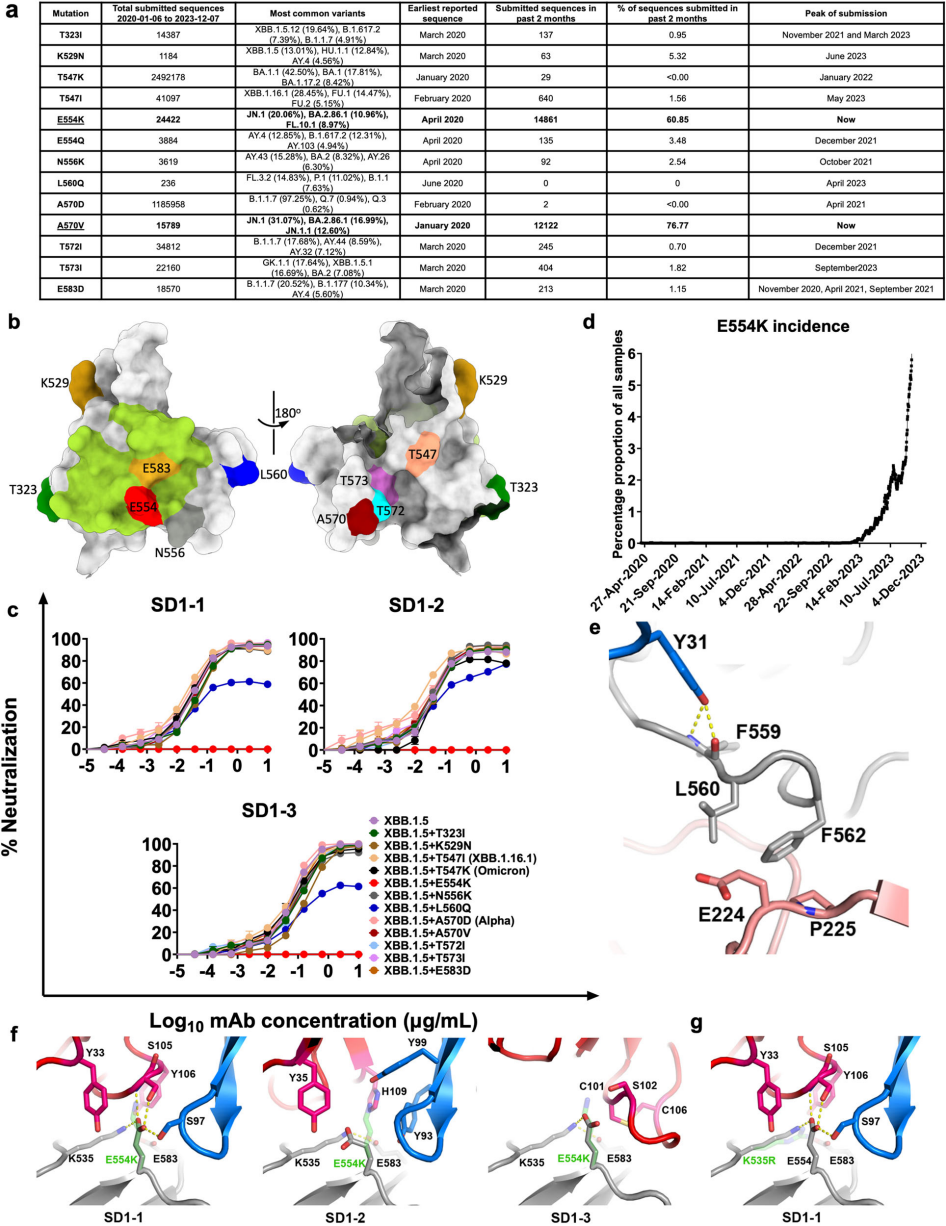
Mutations in SD1 lead to loss of neutralizing activity. **a** Mutations found in SD1 together with their incidence, the most prevalent variants, their first recorded submission, the percentage of sequence reports for each mutation in the last 2 months, and peak time of submission. **b** Front (left panel) and back (right panel) views of the SD1 domain are shown as surface representations with the SD1-1 footprint colored in pale green and mutation sites from **a** highlighted in different colors. **c** Neutralization assays using indicated pseudoviral constructs with mAbs SD1-1, 2, 3, IC50 values are shown in Supplementary Table 4. *n* = 2 independent experiments with duplicates. Data are presented as mean values ± SEM. **d** E554K submitted sequences over time. **e** Residue L560 packs against E224 from NTD of a neighboring chain, it does not have any direct contact with any of the three SD1 Fabs reported here except its proceeding residue F559 H-binds to Y31 of SD1-3 LC. **f** Interactions of E554 with SD1-1, 2, and 3, which are potentially interrupted by E554K mutation (semi-transparent green sticks) in BA.2.86. **g** Effect of K535R on SD1-1 and SD1-3. The drawing and color schemes are as in Fig. 4e. Source data are provided as a Source Data file.

We searched SARS-CoV-2 sequences for mutations occurring in SD1 since the beginning of the pandemic^23^, mutations occurring more than 100 times are shown in Fig. 6a. The positions of mutations on SD1 are shown in Fig. 6b, where the footprint of interaction with SD1-1 mAb is shown, which overlaps mutations E554K and E583D. Mutations were introduced into the XBB.1.5 pseudovirus construct and pseudoviruses containing 12 different mutations in SD1 were then tested in neutralization assays using mAbs SD1-1, −2, −3. Mutations T323I, K529N, T547I, T547K, N556K, L560Q, A570D, A570V, T572I, T573I, and E583D had no effect on neutralization, however, mutation E554K led to complete knock out of activity of mAbs SD1-1, −2, and −3 (Fig. 6c). Mutation L560Q led to a plateau in neutralization titers of around 60%. Finally, we tested neutralization of Alpha and BA.1, which contain mutations A570D and T547K in SD1, using live virus assays, SD1-1, 2, 3, 4 were, as expected from the structural analysis, not affected (Supplementary Fig. 1b)

Mutation E554K was first seen in the B.1 variant from a sequence collected on 13 April 2020 (EPI_ISL_510548) (Fig. 6a) but has been a relatively low-frequency mutation during the pandemic (Fig. 6d). Recently, however, E554K has been increasing in frequency, it is fixed in the recently described BA.2.86 variant (13688 sequences submitted to date), which is believed to have evolved from BA.2 and is causing concern; BA.2.86 completely knocks out neutralization by SD1-1,2,3 (Supplementary Table 4). E554K is also found in FL.10.1 (2222 sequences) and XBB.1.19.1 (638 sequences) and is now emerging in JN.1 (7163 sequences) and JN.4 (176 sequences), two variants from the BA.2.86 lineage with very high growth advantages within the past month, 99% and 55% respectively. It is worth noting, that position 554 is the most variable in the SD1 domain, with 8 different amino acid changes reported to date: K (24422 sequences), G (4442), Q (3884), D (2297), A (1059), V (528), R (27), and N (4).

The structural explanation of why the E554K mutation demolishes the neutralization ability of all three SD1 mAbs reported here is shown in Fig. 6g. In the structures, E554 is stabilized by a salt bridge to K535 of the K535, E554, and E583 triad, and has direct interactions with CDR-H3 of the three SD1 Fabs by either H-binding or salt-bridging (Fig. 4e, g, i). The E554K change will therefore disrupt interactions both within the triad and directly with all the SD1 mAbs. In contrast, the previously identified less potently neutralizing anti-SD1 mAbs SD1.040 and P008-60, which bind higher on SD1 and neutralize by destabilizing the prefusion spike do not appear to form direct interactions with E554 (Supplementary Fig. 2d)^31,32^. Finally, we tested whether the poorly or non-neutralizing mAb isolated during this study were sensitive to E554K by ELISA which demonstrated that only one lost binding to BA.2.86 S, indicating that the poorly or non-neutralizing mAb occupied a different epitope than SD1 mAbs 1–4 (Supplementary Fig. 5a).

## Discussion

Intensive viral sequencing efforts have documented the evolution of SARS-CoV-2 in great detail over the last 4 years. S has been a hotspot for mutation, and while early mutations, such as N501Y in the Alpha variant, may have occurred principally to increase affinity to ACE2 and increase transmissibility^19^, others were likely selected as they lead to evasion of the antibody response allowing infection of vaccinated or previously infected individuals^34,35^.

Within S, mutational hotspots occur in the RBD and NTD, which are the sites for binding of the most potent mAbs isolated from infected or vaccinated volunteers^6,28^. The effects of these mutations have been profound, greatly lowering the neutralization titers of naturally infected or vaccinated serum and leading to the knock-out of activity of almost all mAbs, including those developed for clinical use^36–38^.

Here, we describe an analysis of the antibody response to a separate domain of the spike, SD1, which has been noted before as the target for mAbs, but these mAbs have not been highly potent^31–33^. In our study we also find that the majority of mAbs binding to SD1 show little or no neutralizing activity, but we isolated 4 potent mAbs that each bind to a similar epitope on SD1. Previously characterized anti-SD1 antibodies binding higher on SD1 were found to act by destabilizing the prefusion spike^31,32^. S3H3, which binds an epitope on SH1 similar to the potent anti-SD1 mAbs we identify, and antibodies 12–16 and 12–19, which bind at the interface of SD1 and the NTD, were reported to tighten the S trimer, locking it into the prefusion state^33^. Our more potent antibodies bind to the all-RBD down spike, suggesting that, like these antibodies^18^, they may stabilize this non-ACE2 binding down conformation. Nevertheless, our mAbs and Fabs do not effectively block recombinant S trimer binding to ACE2 in an ELISA-based assay, suggesting that locking down of the RBDs is not the principal mechanism of neutralization. However. when whole virions are used, they can block ACE2 binding. We cannot fully explain the discrepancies between these assay formats, but the ELISA assay may not be a reliable surrogate for the spike conformation in vivo. The increase in neutralization titers afforded by IgG1 vs Fab may represent increased avidity of binding afforded by interaction with two SD1 domains. Such avidity could come from binding two SD1s in a single trimer or from the bridging of trimers. Inspection of the interaction of the potent anti-SD1 mAb described here could not engage 2 SD1 domains on a single spike trimer, whilst bridging adjacent spike trimers could occur, given the flexibility of the hinge in IgG1 (Supplementary Fig. 5b). We note that the perpendicular orientation of the Fab portion on the spike would effectively force the adjacent trimers apart (both as IgG1 and Fab), such that the distance between trimers might be too great to allow dimeric ACE2 to bridge trimers on the cell surface. It has been shown that ACE2 induces oligomerization of spike and that alternate bridging by mAbs can inhibit the virus^39^. It is, therefore, possible that the unusual perpendicular lateral engagement by these potent SD1 binding mAbs leads to neutralization via steric exclusion of ACE2 oligomerization.

Using SD1 depletion assays, we show that anti-SD1 activity comprises a greater fraction of the polyclonal neutralizing response to XBB.1.5 than against early pandemic viruses (the residual high neutralization titers to early pandemic viruses are likely responses to vaccination). The multiple mutations in the NTD and RBD of XBB.1.5 lead to large falls of the neutralization titers of immune sera to XBB.1.5 compared to early pandemic Victoria virus, whilst SD1 remains unmutated in XBB.1.5 and nearly half of the neutralizing activity in XBB.1.5 infected patients is mediated through antibodies targeting SD1. We propose that this increase in prominence of anti-SD1 responses has led to an increasing pressure to select viruses able to evade anti-SD1 antibodies, explaining the development of the E554K mutation in BA.2.86 lineage, XBB.1.19.1 and FL.10.1 variants, which completely knocks out the activity of all four potent SD1 mAbs identified here.

## Methods

### Ethics statement

Our research complies with all relevant ethical regulations. The study protocol was approved by the University of Oxford Central University Research Ethics Committee.

### Bacterial strains and cell culture

Vero (ATCC CCL-81) and VeroE6/TMPRSS2 cells were cultured in Dulbecco’s Modified Eagle medium (DMEM) high glucose (Sigma-Aldrich) supplemented with 10% fetal bovine serum (FBS), 2 mM GlutaMAX (Gibco, 35050061), and 100 U/ml of penicillin–streptomycin at 37 °C. Human mAbs were expressed in HEK293T cells cultured in FreeStyle™ 293 Expression Medium (Cat# 12338018, Gibco™) at 37 °C with 5% CO_2_. HEK293T (ATCC CRL-11268) cells were cultured in DMEM high glucose (Sigma-Aldrich) supplemented with 10% FBS, 1% 100X Mem Neaa (Gibco), and 1% 100X L-Glutamine (Gibco) at 37 °C with 5% CO_2_. BA.5 RBD were expressed in HEK293T (ATCC CRL-11268) cells cultured in FreeStyle™ 293 Expression Medium (Cat# 12338018, Gibco™) at 37 °C with 5% CO_2_. To express other RBD variants and ACE2, HEK293T cells were cultured in DMEM high glucose (Sigma) supplemented with 2% FBS, 1% 100X Mem Neaa, and 1% 100X l-Glutamine at 37 °C for transfection. *E.coli DH5α* bacteria were used for transformation and large-scale preparation of plasmids. A single colony was picked and cultured in LB broth at 37 °C at 200 rpm in a shaker overnight. To produce pseudotyped lentivirus, HEK293T/17 cell was cultured in Dulbecco’s Modified Eagle medium (DMEM) high glucose (Sigma-Aldrich) supplemented with 10% fetal bovine serum (FBS), 2 mM GlutaMAX (Gibco, 35050061), and 100 U/ml of penicillin–streptomycin at 37 °C.

### Sera and PBMC from BA.4/5 infected cases and breakthrough infections in the past 12 months, study subjects

Following informed consent, individuals with omicron BA.4, BA.5, BA.2.73, BA.5.1, BA.5.2, XBB.1.5, BE.1, CH.1.1, CH.1.1.2, and BQ.1.1 were co-enrolled into one or more of the following three studies: the ISARIC/WHO Clinical Characterization Protocol for Severe Emerging Infections [Oxford REC C, reference 13/SC/0149], the “Innate and adaptive immunity against SARS-CoV-2 in healthcare worker family and household members” protocol (approved by the University of Oxford Central University Research Ethics Committee), or the Gastro-intestinal illness in Oxford: COVID sub-study [Sheffield REC, reference16:/YH/0247]. The diagnosis was confirmed through reporting of symptoms consistent with COVID-19, hospital presentation, and a test positive for SARS-CoV-2 using reverse transcriptase polymerase chain reaction (RT-PCR) from an upper respiratory tract (nose/throat) swab tested in accredited laboratories and lineage sequence confirmed through national reference laboratories in the United Kingdom. A blood sample was taken following consent at least 14 days after PCR test confirmation. Clinical information, including severity of disease (mild, severe, or critical infection according to recommendations from the World Health Organization) times between symptom onset and sampling, and age of participant was captured for all individuals at the time of sampling. Sex and gender were not considered in the study design. The sex and gender of participants were determined based on self-report.

### Isolation of BA.4/5S-specific and SD1-specific single B cells by FACS

SD1-1 was isolated from a BA.4/5S-specific single B cell sort. PBMC were stained with LIVE/DEAD Fixable Aqua dye (Invitrogen) followed by recombinant trimeric S-twin-Strep of BA.4/5. Cells were then incubated with CD3-FITC (7.5 μl in 100 μl sorting buffer, purchased from BD, #555332), CD14-FITC (7.5 μl in 100 μl sorting buffer, purchased from BD, #555397), CD16-FITC (7.5 μl in 100 μl sorting buffer, purchased from BD, #555406), CD56-FITC (1.5 μl in 100 μl sorting buffer, purchased from BD, #562794), IgM-FITC (7.5 μl in 100 μl sorting buffer, purchased from BD, #555782), IgA-FITC (1.5 μl in 100 μl sorting buffer, purchased from Dako, #F0188), IgD-FITC (1.5 μl in 100 μl sorting buffer, purchased from Dako, #F0189), IgG-BV786 (1.5 μl in 100 μl sorting buffer, purchased from BD, #564230) and CD19-BUV395 (1.5 μl in 100 μl sorting buffer, purchased from BD, #563549), along with Strep-MAB-DY549 (1.5 μl in 100 μl sorting buffer, purchased from iba, #2-1566-050) to stain the twin-strep tag of the S protein. IgG^+^ memory B cells were gated as CD19^+^, IgG^+^, CD3^−^, CD14^−^, CD56^−^, CD16^−^, IgM^−^, IgA^−^, and IgD^−^, and S^+^ was further selected, and single cells were sorted into 96-well PCR plates with 10 µl of catching buffer (Tris, Nuclease free-H_2_O and RNase inhibitor). Plates were briefly centrifuged at 2000×*g* for 1 min and left on dry ice before being stored at −80 °C.

SD1-specific single B cells were sorted the same way as BA.4/5S-specific B cells, except for exchanging S protein with SD1-6×His, and 6×His-tag was stained by anti-His-PE (1.5 μl in 100 μl sorting buffer, purchased from BioLegend, #362603).

### Cloning and expression of human mAbs

SD1-specific human mAbs were cloned and expressed as described previously^6^. Briefly, genes for Ig IGHV, Ig Vκ, and Ig Vλ were recovered from positive wells by RT-PCR. Genes encoding Ig IGHV, Ig Vκ, and Ig Vλ were then amplified using Nested-PCR by a cocktail of primers specific to human IgG. PCR products of HCs and LCs were ligated into the expression vectors of human IgG1 or immunoglobulin κ-chain or λ-chain by Gibson assembly^40^. For mAb expression, plasmids encoding HCs and LCs were co-transfected by PEI-transfection into a HEK293T cell line, and supernatants containing mAbs were collected and filtered 4-5 days after transfection, and the supernatants were further characterized or purified.

### ACE2-spike interaction inhibition assay by ELISA

MAXISORP immunoplates were coated with 5 µg/ml of purified ACE2-His protein overnight at 4 °C and then blocked by 2% BSA in PBS. Meanwhile, mAbs were serially diluted and mixed with 2.5 µg/ml of recombinant XBB.1.5 trimeric S-twin-Strep. Antibody-S protein mixtures were incubated at 37 °C for 1 h. After incubation, the mixtures were transferred into the ACE2-coated plates and incubated for 1 h at 37 °C. After wash, Strep-MAB-Classic (2-1507-001, IBA) was diluted at 0.2 μg/ml by 2% BSA and used as the primary antibody, followed by Goat anti-mouse IgG-AP (A9316, Sigma-Aldrich) at 1:10,000 dilution. The reaction was developed by adding PNPP substrate and stopped with NaOH. The absorbance was measured at 405 nm. The ACE2/S binding inhibition was calculated by comparing it to the antibody-free control well. IC50 was determined using the PROBIT program from the SPSS package.

### mAb-spike interaction inhibition by ACE2

MAXISORP immunoplates were coated with 5 μg/mL of Wuhan S. After blocking with 2% BSA, serial diluted ACE2-mouse Fc, starting from 80 μg/mL with fourfold dilution, were added into each plate. After 1 h incubation at 37 °C, plates were washed with PBST, and 5 μg/mL of SD1 mAbs or mAb222 were added into the plates and incubated for 1 h at 37 °C, followed by washing with PBST. To detect the binding of antibodies with S, anti-human IgG-AP (1:10,000 dilution, A9544, Sigma-Aldrich) was added after wash, incubated for 1 h at 37 °C, and developed by PNPP. To detect the binding of ACE2-mouse Fc on S, the same assay was performed, except that anti-mouse IgG-AP (1:10,000 dilution, A9316, Sigma-Aldrich) was added instead of anti-human IgG-AP after adding SD1 mAb or mAb222, and plates were further developed by PNPP.

### ACE2-live virus interaction inhibition assay by ELISA

To measure the ability of inhibition of ACE2-live virus interaction by mAbs, MAXISORP immunoplates were coated with 5 µg/ml of purified mAb166^6^ overnight at 4 °C and then blocked by 2% BSA in PBS. Live virus XBB.1.5 was diluted in 2% BSA to 1 × 10^5^ FFU/mL, and 100 μL of virus dilution was added into each well. After 1 h of incubation at 37 °C, plates were washed by PBST, serial diluted mAbs were added, and plates were incubated at 37 °C for an hour. Then 5 μL of 100 μg/mL ACE2-mouse Fc was added into each well without washing, and anti-mouse IgG-AP (1:10,000 dilution, A9316, Sigma-Aldrich) was added after 1 h of incubation at 37 °C and PBST washing, followed by adding PNPP substrate and stopped with NaOH. The absorbance was measured at 405 nm. The ACE2/Live virus interaction inhibition was calculated by comparing it to the antibody-free control well. IC50 was determined using the PROBIT program from the SPSS package.

### Pseudovirus plasmid construction and lentiviral particle production

Pseudotyped lentivirus expressing SARS-CoV-2 S proteins from ancestral strain (Victoria, S247R), BA.1, BA.1.1, BA.2, BA.2.12.1, BA.2.75, BA.4/5, BA.4.6, BA.2.75.2, BA.2.3.30, BA.2.10.4, BQ.1, BQ.1.1, BS.1, BF.1, BJ.1, BN.1, CH.1.1, CA.3.1, XBB, XBB.1, and XBB.1.5 were constructed as described previously^7,41–43^. We applied the same method to construct XBB.1.5.10 and XBB.1.5.70, by adding more mutations into the XBB.1.5 construct. To generate XBB.1.5.10, we added the F456L mutation, and to create XBB.1.5.70, we added L455F into the XBB.1.5.10 backbone. Single mutations in the SD1 site were introduced using the same method into the XBB.1.5 backbone. SD1 mutations, which were introduced are: T323I, K529N, T547I, T547K, E554K, N556K, L560Q, A570D, A570V, T572I, T573I, and E583D. Plasmid to create BA.2.86 PV was custom-synthesized by Integrated DNA Technologies based on the wild-type SARS-CoV-2 BA.2.86 (EPI_ISL_18110065) and cloned into pcDNA3.1 plasmid. This plasmid carries the S gene and was used for generating pseudoviral particles together with the lentiviral packaging vector and transfer vector encoding luciferase reporter. BA.2.86 plasmid containing the following mutations was produced: ins16MPLF, T19I, R21T, L24del, P25del, P26del, A27S, S50L, H69del, V70del, V127F, G142D, Y144del, F157S, R158G, N211del, L212I, V213G, L216F, H245N, A264D, I332V, G339H, K356T, S371F, S373P, S375F, T376A, R403K, D405N, R408S, K417N, N440K, V445H, G446S, N450D, L452W, N460K, S477N, T478K, N481K, V483del, E484K, F486P, Q498R, N501Y, Y505H, E554K, A570V, D614G, P621S, H655Y, N679K, P681R, N764K, D796Y, S939F, Q954H, N969K, and P1143L. To generate JN.1 and JN.4, we introduced the L455S and A475V mutations, respectively, using primers. The resulting pcDNA3.1 plasmid carrying S gene was used for generating pseudoviral particles together with the lentiviral packaging vector and transfer vector encoding luciferase reporter. All the constructs were Sanger sequence confirmed.

### Pseudoviral neutralization test

The pseudoviral neutralization test has been described previously^42^. Briefly, the neutralizing activity of potent SD1 antibodies generated from donors who had recovered from breakthrough infections were tested against Victoria, Alpha, BA.1, BA.1.1, BA.2, BA.2.12.1, BA.2.75, BA.2.75.2, BA.2.3.20, BA.2.10.4, BJ.1, BA.4/5, BA.4.6, BQ.1, BQ.1.1, BS.1, BF.7, BN.1, XBB, XBB.1, XBB.1.5, XBB.1.5.10, XBB.1.5.70, CH.1.1, CA.3.1, BA.2.86, JN.1, and JN.4, and the neutralizing activity of potent SD1 antibodies was also tested against XBB.1.5 with single mutations in SD1 site: T323I, K529N, T547I, T547K, E554K, N556K, L560Q, A570D, A570V, T572I, T573I, and E583D. Fourfold serial diluted mAbs were incubated with pseudoviral particles at 37 °C with 5% CO_2_ for 1 h. Stable HEK293T/17 cells expressing human ACE2 were then added to the mixture at 1.5 × 10^4^ cells/well. Forty-eight hours post-infection, culture supernatants were removed, and 50 μL of 1:2 Bright-Glo TM Luciferase assay system (Promega, USA) in 1 × PBS was added to each well. The reaction was incubated at room temperature for 5 min and firefly luciferase activity was measured using CLARIOstar® (BMG Labtech, Ortenberg, Germany). The percentage neutralization was calculated relative to the control. Probit analysis was used to estimate the dilution that inhibited half maximum pseudotyped lentivirus infection (PVNT50).

### Focus reduction neutralization assay (FRNT)

The neutralization potential of Ab was measured using a focus reduction neutralization test (FRNT), where the reduction in the number of infected foci is compared to a negative control well without antibodies. Briefly, serially diluted Ab or plasma was mixed with SARS-CoV-2 strains and incubated for 1 h at 37 °C. The mixtures were then transferred to 96-well, cell culture-treated, flat-bottom microplates containing confluent Vero cell monolayers in duplicate and incubated for a further 2 h, followed by the addition of 1.5% semi-solid carboxymethyl cellulose (CMC) overlay medium to each well to limit virus diffusion. A focus forming assay was then performed by staining Vero cells with human anti-NP mAb (mAb206, produced in-house) followed by peroxidase-conjugated goat anti-human IgG (A0170; Sigma). Finally, the foci (infected cells), approximately 100 per well in the absence of antibodies, were visualized by adding TrueBlue Peroxidase Substrate. Virus-infected cell foci were counted on the classic AID ELISpot reader using AID ELISpot software. The percentage of focus reduction was calculated, and IC_50_ was determined using the PROBIT program from the SPSS package.

### Plasma anti-SD1 antibody depletion assay

To deplete the anti-SD1 antibody, SD1 with Twin-strep tag was conjugated with strep-Tactin beads (2-5030-010, IBA) overnight at 4 °C. Conjugated beads were then incubated with 200 μL of plasma of interest at dilution of 1:5, and beads incubated in the absence of SD1 antigen were used as a beads-only, mock control. After overnight incubation, beads were cleaned out by centrifuging at 11,000×*g* for 5 min, and the remaining depleted samples were collected, filter sterilized, and tested for complete depletion by ELISA.

### Cloning of spike, RBD, NTD, and SD1

Expression plasmids encoding Wuhan spike^6^, BA.4 spike, BA.4 NTD, and RBD were constructed with human codon-optimized sequence^43^. To create BA.2.12.1 spike, spike sequence was amplified from BA.2.12.1 pseudovirus expression plasmid^4^ and ligated into pHLsec vector with a T4 fibritin trimerization domain, an HRV 3C cleavage site, a His-8 tag and a Twin-Strep-tag at the C terminus, resulting the spike without RRAR to GSAS (aa 682–685) and KV to PP (aa 986–987) mutations. Mutations of G252V, R346T, L368I, V445P, G446S, N460K, F486P, and F490S were introduced into BA.2 expression plasmids, which we constructed previously^3^, to create XBB.1.5 spike. Expression plasmid of BA.2.86 spike was constructed encoding for human codon-optimized sequences from wild-type SARS-CoV-2 (MN908947) and BA.2.86 (EPI_ISL_18110065). Fragments were cloned in pHLsec vectors downstream of the chicken β-actin/rabbit β-globin hybrid promoter and followed by a T4 fibritin trimerization domain, an HRV 3C cleavage site, a His-8 tag and a Twin-Strep-tag at the C terminus^44^. Mutations coding for stabilizing proline residues and to eliminate putative furin cleavage sites were inserted in BA.2.86 sequence as follows: RRAR > GSAS (aa 682–685) and KV > PP (aa 986–987). Spike includes following mutations: ins16MPLF, T19I, R21T, L24del, P25del, P26del, A27S, S50L, H69del, V70del, V127F, G142D, Y144del, F157S, R158G, N211del, L212I, V213G, L216F, H245N, A264D, I332V, G339H, K356T, S371F, S373P, S375F, T376A, R403K, D405N, R408S, K417N, N440K, V445H, G446S, N450D, L452W, N460K, S477N, T478K, N481K, V483del, E484K, F486P, Q498R, N501Y, Y505H, E554K, A570V, D614G, P621S, H655Y, I670V, N679K, P681R, N764K, D796Y, S939F, Q954H, N969K, and P1143L. Spike fragments were custom-synthesized by Integrated DNA Technologies and cloned into a pHLsec vector. Spike sequence was verified by Sanger sequencing.

The recombinant SD1 protein comprises amino acids 320 V to 331 N of the spike, a GGSGGSGGS linker, and amino acids 528 K to 591 S of the spike with a double strep tag at the C terminus for purification. The constructs were verified by Sanger sequencing.

### Protein production

Protein expression and purification were largely the same as described previously^6,34^. Twin-strep tagged Wuhan, BA.2.12.1, BA.4, XBB.1.5, and BA.2.86 spikes were transiently expressed in HEK293T cells and purified with Strep-Tactin XT resin (IBA Lifesciences). Plasmids encoding BA.4 RBD, NTD, and SD1 with a 6*His-tag were separately transiently expressed in Expi293F™ Cells (Thermo Fisher), cultured in FreeStyle™ 293 Expression Medium (Thermo Fisher) at 30 °C with 8% CO_2_ for 4 days. The harvested medium was concentrated and buffer-exchanged using a QuixStand benchtop system. His-tagged proteins were purified with a 5 mL HisTrap nickel column (Cytiva), followed by a Superdex 75 10/300 GL gel filtration column (Cytiva).

### IgG mAbs and Fabs production

For the in-house antibodies, heavy and light chains of the indicated antibodies were transiently transfected into 293T cells, and antibody purified from supernatant on protein A^7^. Fabs were digested from purified IgGs with papain using a Pierce Fab Preparation Kit (Thermo Fisher), following the manufacturer’s protocol.

### Competition assay of BA4/5-2 and SD1-1

This competition assay was performed on a Fortebio Octet RED96e machine with Fortebio Anti-HIS (HIS2) Biosensors. 2 μg ml^−1^ of His-tagged Wuhan spike dissolved in the running buffer (10 mM HEPES, pH 7.4, and 150 mM NaCl) was used as the ligand and was first immobilized onto the biosensors. The biosensors were then washed in the running buffer to remove unbound spike. Each biosensor was dipped into saturating antibodies (Ab1) to saturate the bound spike, except one biosensor was into the running buffer in this step, acting as the reference. The concentration of saturating antibodies used was 15 μg ml^−1^. Then all biosensors were washed with the running buffer again and dipped into wells containing the same competing antibody (Ab2). The concentration of competing antibodies used was 5 μg ml^−1^. The y-axis values of signals from different biosensors in this step were divided by the value of the reference channel to get the ratio of results for different Ab1-Ab2 pairs. A ratio close to 0 indicated total competition, while 1 indicated no competition.

### Cryo-EM methods

Complexes were prepared as close to blotting as possible. Each SD1 Fab was added at a sixfold molar excess (twofold excess of sites, assuming three sites per Spike protein, 0.3 mg/mL final concentration of S) to either prefusion stabilized BA.4 spike, which contains 2 P mutation and deletion of furin cleavage site (SD1-1), or BA.2.12.1 (SD1-2 and SD1-3) spike, which retains the native sequence, and immediately applied to C-flat 2/1 200 mesh copper grids (Protochips), blotted for 5 s (force −1, Vitrobot Mark IV) and vitrified in liquid ethane. Movies were collected in EER format using EPU on a Titan Krios operating at 300 kV with a Falcon4i/SelectrisX (Supplementary Table 1), with 50 EER fractions and no oversampling. Data were binned four times and pre-processed in the CryoSPARC Live interface, with downstream processing performed using CryoSPARC™^45,46^.

For SD1-1, an initial set of 901,138 particles were template picked on the fly and 2D classified into 150 groups. The most promising classes, bearing secondary structural detail and a variety of views (137,816 particles) were selected and subjected to heterogeneous refinement (no symmetry) using three template volumes generated ab initio from a subset of 205,090 particles. The best class, with 108,365 particles, comprised a volume clearly decorated with three Fabs, and all RBDs in the downwards conformation, adhering to C3 symmetry. This particle set was non-uniform refined with C3 symmetry before un-binning and further non-uniform refinement^47^, resulting in a reconstruction to 3.1 Å resolution (gold-standard FSC^48^ in CryoSPARC™) (Supplementary Fig. 3). Following two rounds of global/local CTF refinement, the resulting resolution was 2.7 Å (sharpened with a −70.2 Å^2^ B-factor).

For SD1-2, 162,416 picked on-the-fly particles were 2D classified into 95 templates, and 21,480 particles were used to generate three ab initio models, which were then used as reference volumes for heterogeneous refinement and the class of this subset with trimeric spike clearly decorated with three fabs was used as a reference model for the complete set of particles. Particles were then picked from all micrographs, 764,482 in total, and classified into 250 2D classes. About 506,421 particles across 66 classes were then used for refinement with reference volumes generated from the subset described above as an initial volume showing a spike clearly decorated with three Fabs (503,734 particles). Particles were non-uniform and refined with C3 symmetry before extraction from the 9486 micrographs. Further non-uniform refinement yielded a reconstruction at 2.3 Å. CTF refinement (tilt, trefoil, spherical aberration, tetrafoil, and anisotropic magnification fitted) and a second round of non-uniform refinement, yielded a final reconstruction at 2.2 Å^48^ (Supplementary Fig. 3) also with C3 symmetry, B-factor -68.0 Å^2^ which was then used for refinement.

For SD1-3, particle picking was performed using the Blob-picker module, and the initial extracted set (3,282,771 particles, binned four times) were 2D classified into 250 classes. From this, 478,172 particles were selected from 14 classes, representing a good sampling of views of Spike. This set was then further classified, in 3D with C3 symmetry via heterogeneous refinement, using three ab initio models generated from a subset of 100,000 particles generated on-the-fly, with one ab initio model already clearly showing Spike in an “all-down” RBD configuration encircled by three Fabs. This initial 3D set of 409,222 particles was non-uniform refined with C3 symmetry, resulting in a reconstruction at Nyquist (3.0 Å, −66.6 Å^2^ B-factor). Further 3D classification was performed, without alignment, focussed around one Fab, NTD, RBD and S1. The classes with clear Fab decoration (167,816 particles, 4/10 classes) were pooled, unbinned, and used non-uniform refinement with C3 symmetry, to yield a final reconstruction at 2.2 Å^48^ (Supplementary Fig. 3).

Modeling^49^ used PDB:8CIM as an initial basis. For the Fab variable domains, the top BLAST^50^ hits for the H and L chain sequences of SD1-3 were used as an initial model and adjusted accordingly. For SD1-2, refinement was performed using the SD1-3 model as an initial basis. Refinement/model building used Coot and Phenix^51,52^.

### Statistical and reproducibility

For the SD1 antibody depletion assay, geometric means are shown in each column and Wilcoxon matched-pairs signed-rank test was used for the analysis, and two-tailed *P* values were calculated. No statistical method was used to predetermine the sample size. No data were excluded from the analyses. The experiments were not randomized. The Investigators were not blinded to allocation during experiments and outcome assessment.

### Reporting summary

Further information on research design is available in the Nature Portfolio Reporting Summary linked to this article.

### Supplementary information

Supplementary Information

Peer Review File

Reporting Summary

### Source data

Source Data

## Data Availability

The data that support this study are available from the corresponding author upon request. The atomic models and cryo-EM density maps have been deposited into the Protein Data Bank (PDB) and Electron Microscopy Data Bank (EMDB) as follows: BA.4-spike/SD1-1 (AKA BA.4/5-5): PDB 8CIN and EMD-16680, BA.2.12.1-spike/SD1-2: PDB 8R1C and EMD-18807, BA.2.12.1-spike/SD1-3: PDB 8R1D and EMD-18808. Previously published: BA.2-07 Fab in complex with SARS-COV-2 BA,2,12,1 spike glycoprotein: PDB 8CIM. Source data are provided with this paper.
