# Supplementary material for: The SARS-CoV-2 neutralizing antibody response to SD1 and its evasion by BA.2.86
Source: Nat Commun. 2024 Mar 28;15:2734. doi: 10.1038/s41467-024-46982-6 (PMC10978878; doi:10.1038/s41467-024-46982-6)
Supplement: Supplementary file 1 — Supplementary Information [file 41467_2024_46982_MOESM1_ESM.pdf]

The SARS-CoV-2 neutralizing antibody response to SD1 and its evasion by BA.2.86

Daming Zhou[1,2,3,#], Piyada Supasa[1,4,#], Chang Liu[1,4,#], Aiste Dijokaite-Guraliuc[4,#], Helen M.E. Duyvesteyn[2,#], Muneeswaran Selvaraj[4], Alexander J. Mentzer[4,5], Raksha Das[4], Wanwisa Dejnirattisai[6], Nigel Temperton[7], Paul Klenerman[5,8,9], Susanna J. Dunachie[5,10,11], Elizabeth E. Fry[2,*], Juthathip Mongkolsapaya[1,4,11,*], Jingshan Ren[2,*], David I. Stuart[1,2,12,*,^], Gavin R Screaton[1,4,*]

1. Chinese Academy of Medical Science (CAMS) Oxford Institute (COI), University of Oxford, Oxford, UK

2. Division of Structural Biology, Nuffield Department of Medicine, University of Oxford, Centre for Human Genetics, Oxford, UK.

3. College of Life Sciences, Zhejiang University, Hangzhou 310058, China.

4. Centre for Human Genetics, Nuffield Department of Medicine, University of Oxford, Oxford, UK

5. NIHR Oxford Biomedical Research Centre, Oxford University Hospitals NHS Foundation Trust, Oxford, UK.

6. Division of Emerging Infectious Disease, Research Department, Faculty of Medicine Siriraj Hospital, Mahidol University, Bangkok-Noi, Bangkok 10700, Thailand

7. Viral Pseudotype Unit, Medway School of Pharmacy, University of Kent and Greenwich Chatham Maritime, Kent ME4 4TB, UK

8. Peter Medawar Building for Pathogen Research, University of Oxford, Oxford, UK

9. Translational Gastroenterology Unit, Nuffield Department of Medicine, University of Oxford, Oxford, UK

10. NDM Centre For Global Health Research, Nuffield Department of Medicine, University of Oxford, Oxford, UK

11. Mahidol-Oxford Tropical Medicine Research Unit, Bangkok, Thailand

12. Diamond Light Source Ltd, Harwell Science & Innovation Campus, Didcot, UK

# These authors contributed equally to this work.

* Corresponding authors: liz@strubi.ox.ac.uk, juthathip.mongkolsapaya@well.ox.ac.uk, ren@strubi.ox.ac.uk, dave@strubi.ox.ac.uk, gavin.screaton@medsci.ox.ac.uk

^ Lead contact

a

BA4/5-2 used for saturating, SD1-1 for subsequent binding

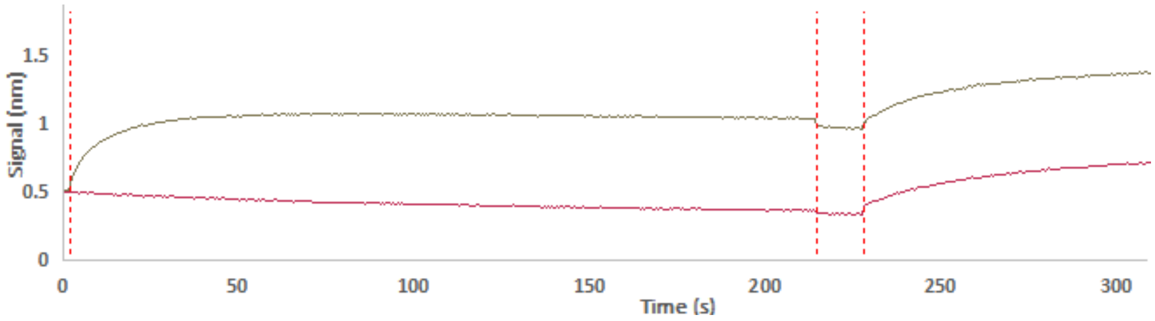

b

SD1-1 used for saturating, BA4/5-2 for subsequent binding

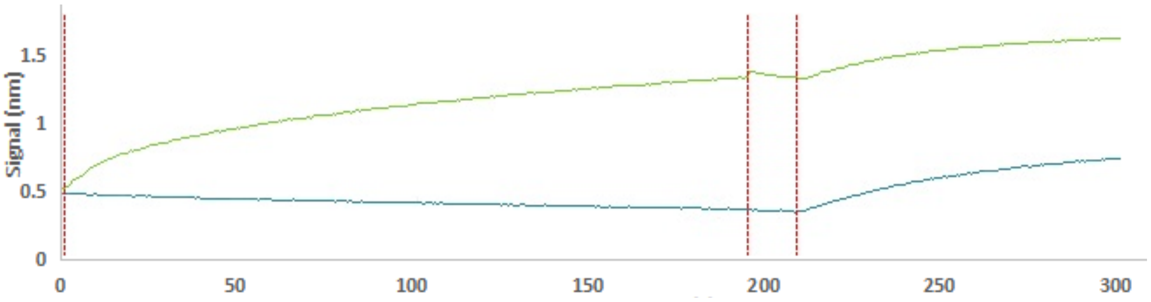

c

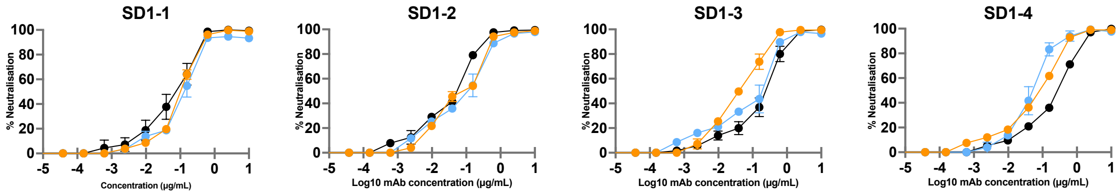

d

Live virus-IC50 (μg/mL)			
	**Victoria**	**Alpha**	**BA.1**
**SD1-1**	0.068 ± 0.009	0.093 ± 0.006	0.049 ± 0.000
**SD1-2**	0.063 ± 0.000	0.067 ± 0.004	0.030 ± 0.004
**SD1-3**	0.038 ± 0.005	0.069 ± 0.007	0.136 ± 0.031
**SD1-4**	0.052 ± 0.008	0.051 ± 0.005	0.170 ± 0.003

Supplementary Figure 1. BLI Competition Assay

a, BLI competition data for BA4/5-2 and SD1-1. Wuhan S was coated onto biosensors and BA4/5-2 (ACE2 blocking anti-RBD mAb) was used first for saturating and SD1-1 mAb used for subsequent binding. **b**, Reverse order, SD1-1 used for saturating and BA4/4-2 for subsequent binding. **c**, **d**, Live virus neutralization assays of Victoria Alpha (A570D) and BA.1 (T547K). Source data are provided as a source data file.

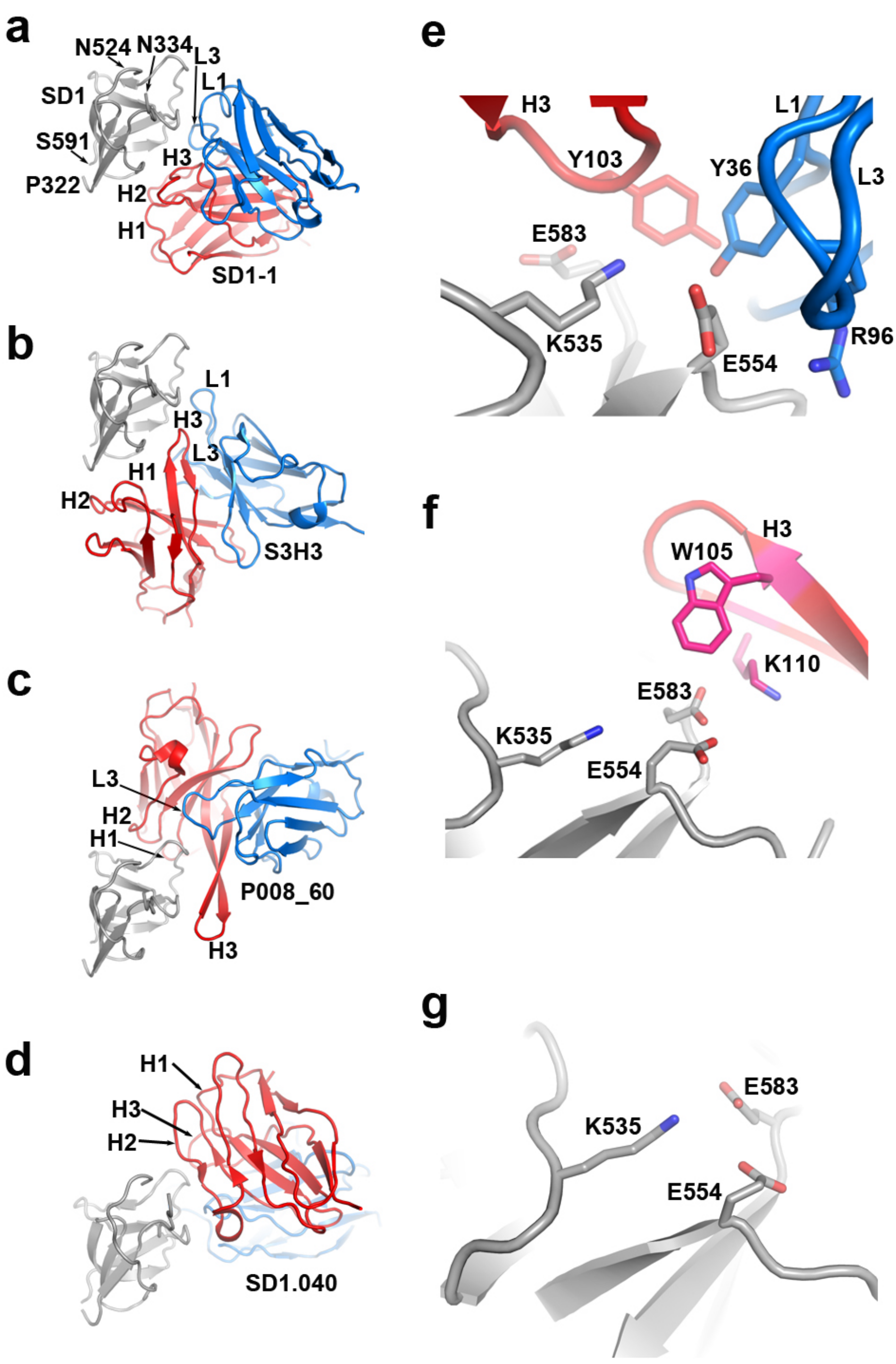

Supplementary Figure 2. Comparison of binding modes of SD1 binding mAbs

a-d, Binding mode comparison of SD1-1 (**a**) with the reported SD1 binders S3H3 (**b**, PDB, 7WD8), P008_60 (**c**, PDB, 7ZBU), SD1.040 (**d**, PDB, 8D48) by overlapping the SD1. **e**, Contacts of SD1 E554 with S3H3[1]. **f**, E554 does not have any contact with P008_60[2], **g** none of the triad SD1-1 residues has contact with SD1.040[3]. Source data are provided as a source data file.

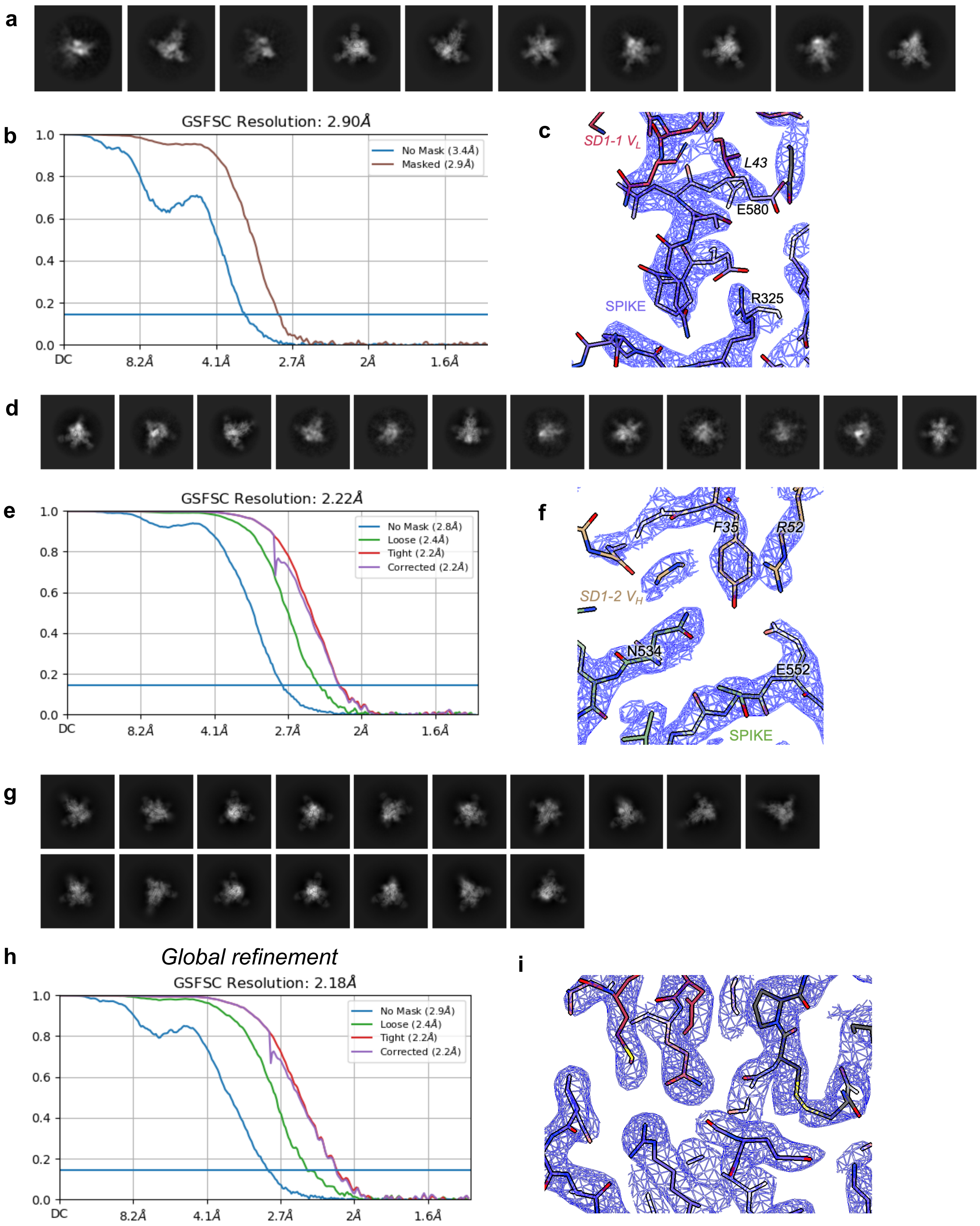

Supplementary Figure 3. Cryo-EM results

a, SD1-1 2D class averages selected for further refinement. **b**, Gold standard FSC plot for final reconstruction (C3 symmetry, generated using CryoSPARCv.4). FSC = 0.143 is shown with a blue horizontal line. **c**, Representative density at the Fab/Spike interface. Residue labels associated with Fab are in italic. The spike model is shown in purple, SD1-1 variable heavy chain in grey, and variable light chain in pink. **d**, SD1-2 2D class averages selected for further refinement. **e**, Gold standard FSC plot for final reconstruction as in **b**. **f**, Representative density at the Fab/Spike interface. The spike model is shown in green, SD1-2 heavy variable chain in sand, and variable light chain in terracotta. Residue labels associated with Fab are in italic. **g**, SD1-3 2D class averages selected for further refinement. **h**, Gold-standard (GS) FSC plot for SD1-3 (prepared using CryoSPARCv4). FSC = 0.143 is shown with a blue horizontal line. **i**, Representative density at the Fab/Spike interface. Residue labels associated with Fab are in italic. The spike model is shown in purple, SD1-3 heavy variable chain in grey, and variable light chain in pink.

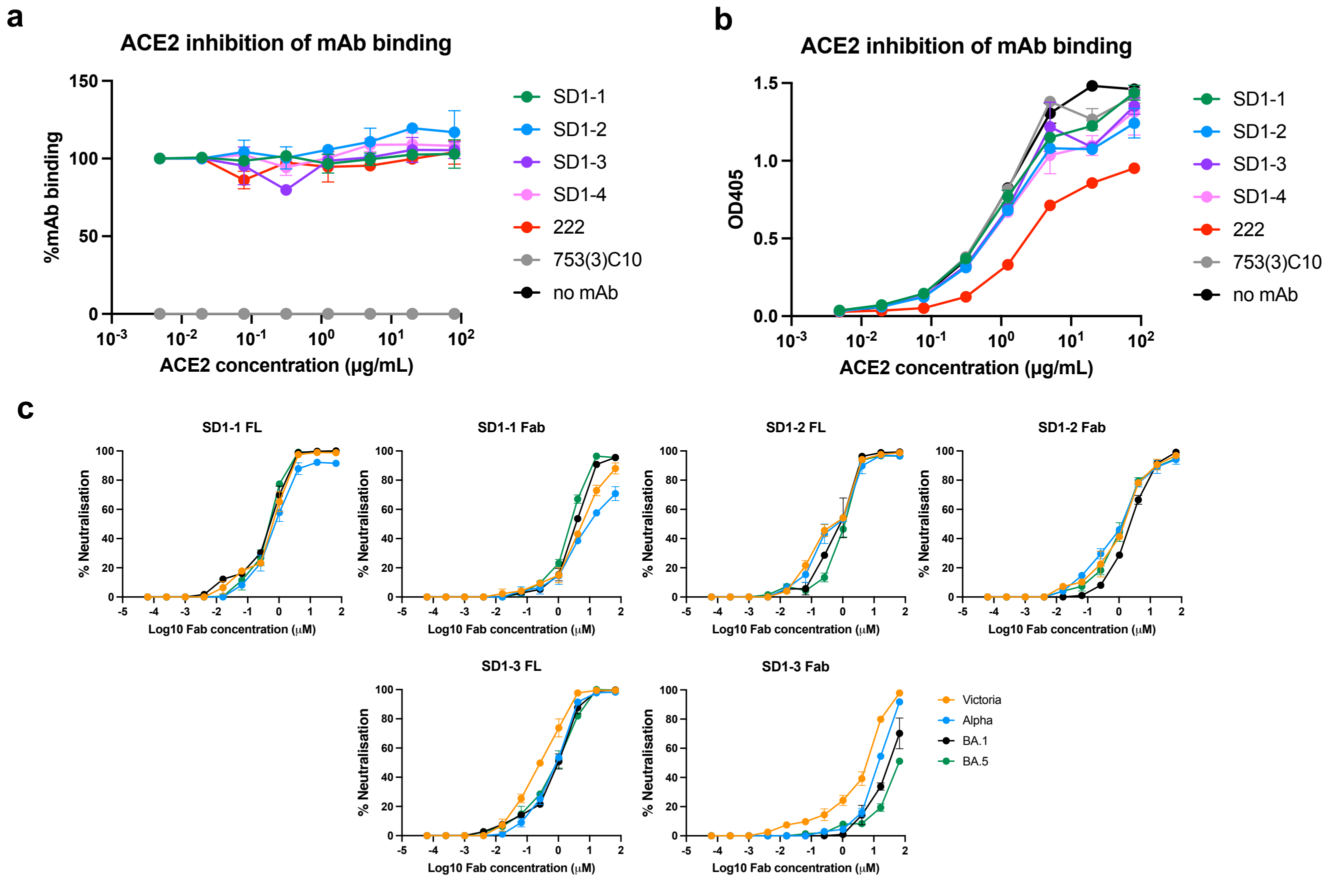

IC50 (µM)						
	SD1-1 FL	SD1-1 Fab	SD1-2 FL	SD1-2 Fab	SD1-3 FL	SD1-3 Fab
Victoria	0.888 ± 0.102	16.384 ± 0.168	0.413 ± 0.003	1.078 ± 0.140	0.252 ± 0.031	2.977 ± 0.129
Alpha	1.473 ± 0.082	33.946 ± 6.764	0.502 ± 0.046	1.000 ± 0.129	0.728 ± 0.077	12.676 ± 0.940
BA.1	0.645 ± 0.003	4.036 ± 0.040	0.594 ± 0.075	2.220 ± 0.018	0.613 ± 0.005	29.951 ± 4.656
BA.5	0.847 ± 0.115	3.755 ± 1.147	0.854 ± 0.112	1.257 ± 0.012	0.606 ± 0.086	>66.7

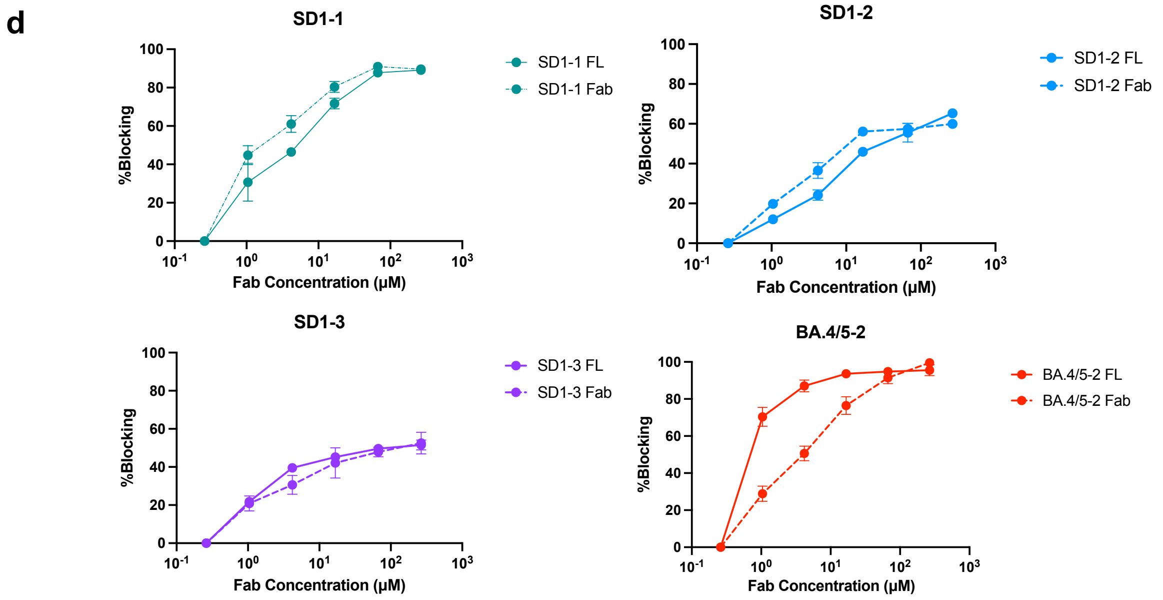

Supplementary Figure 4. ACE2 blocking by SD1 mAbs

a-b, Serially diluted ACE2 was bound to S coated on ELISA plates prior to the addition 5 µg/mL of mAbs. **a,** The percentage of mAb binding to S was calculated relative to mAb binding in wells without ACE2, **b**, the binding of ACE2 to S in the same assay is shown in. mAb222, which is a potent blocker of ACE2-S interaction, was used as a positive control and anti-dengue envelope protein mAb 753(3)C10, which does not block ACE2-S interaction, was used as a negative control. All SD1 mAb bind S complexed with ACE2 but mAb 222 displaces ACE2 upon binding to recombinant S. **c**, Live virus neutralization assays of Victoria, Alpha, BA.1, and BA.5 by Fab full-length (FL) IgG1 anti-SD1 mAbs. IC50 are shown below the neutralization curves representing the molar concentration of Fab (IgG1 contains 2 Fab). All assays have been done with two biologically independent experiments. **d**, Blocking of ACE2 binding to XBB.1.5 virion S by full length and Fab of anti-RBD mAb BA.4/5-2 and anti-SD1 mAb SD1-1, SD1-2, and SD1-3.

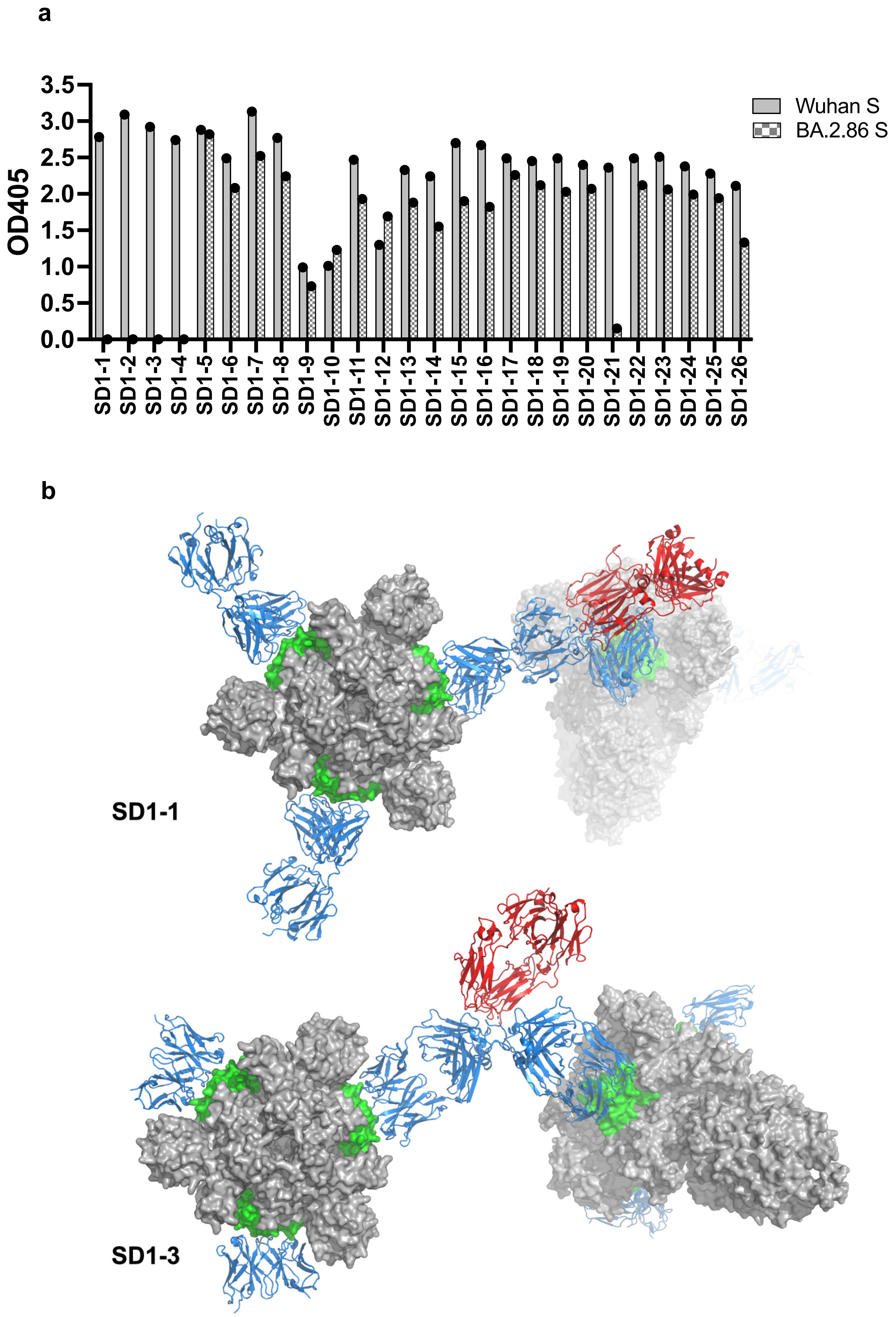

Supplementary Figure 5. ELISA binding to Wuhan and BA.2.86 spike and bridging of spikes by SD1 mAbs

a, Comparison of binding of potent and impotent SD1 mAbs on Wuhan spike and BA.2.86 spike by ELISA, n=1 experiment. **b**, Models of SD1-1 and SD1-3 Mab bridging adjacent spikes. Rotation of a SD1-3 Fab is required to link 2 spikes. Note that the flexibility of the antibody hinge region would mean that bridging could occur with both spikes anchored in the virus membrane. For modelling the SD1 Fabs variable domains were superimposed on a complete IgG (PDB code: 5DK3). Source data are provided as a source data file.

Structure	BA.4-spike/SD1-1	BA.2.12.1-Spike/SD1-2	BA.2.12.1-Spike/SD1-3
PDB/EMDB ID	8CIN/EMD-16680	8R1C/EMD-18807	8R1D/EMD-18808
**Data collection**			
Voltage (kV)	300	300	300
Frames (EER fractions)	50	50	50
Dose rate ($e^-/Å^2/s$)	11.7	17.2	17.9
Total dose ($e^-/Å^2$)	50	50	50
Calibrated pixel size ($Å^2$)	0.7303	0.7303	0.7303
Defocus (μm)	0.8-2.6	0.8-2.6	0.8-2.6
Movies	10,383	9486	10,197
Particles (final)	108,365	503,754	190,797
Symmetry	C3	C3	C3
Map resolution (Å)	2.7	2.2	2.2
Sharpening B-factor ($Å^2$)	-70.2	-68.0	-53.8
**Refinement**			
Resolution (Å)	2.7	2.2	2.3
No. protein atoms	35,187	30,456	30,162
B factors ($Å^2$)	132	59.4	38.3
r.m.s. deviations			
Bond lengths (Å)	0.006	0.004	0.005
Bond angles (°)	0.6	0.7	0.6
Clash score	5.2	6.6	3.6
Ramachandran outlier (%)	0	0	0
Rotamer outlier (%)	1.45	0.15	0.91
d FSC model (0.5)	3.0	2.4	2.1
CC (mask)	0.87	0.84	0.86

Supplementary Table 1. Data collection, structure determination and refinement statistics

Ab id.	Protein-Specific	Heavy chain					Light chain				
		V-GENE and allele	V-REGION Nb of AA changes	J-GENE and allele	D-GENE and allele	CDR3	Light Chain	V-GENE and allele	V-REGION Nb of AA changes	J-GENE and allele	CDR3
SD1-1	SD1	4-59*01 F, or 4-61*01 F	10	4*02 F	1-26*01 F	ARDSVWYTGSYGLIY	λ	2-23*02 F	6	2*01 F, or 3*01 F	CSYAGSSTVV
SD1-2	SD1	4-61*02 F	13	3*02 F	2-15*01 F	VRLDNCSAGYCHAFDI	λ	2-23*02 F	4	1*01 F	CSYAGSGTYV
SD1-3	SD1	3-23*04	6	4*02 F	2-15*01 F	ATHYCSGGSCPFDY	λ	3-1*01 F	12	3*02 F	QAWDSNTAV
SD1-4	SD1	3-23*04	5	4*02 F	2-15*01 F	ATHYCSGGSCPFDY	λ	3-1*01 F	11	3*02 F	QAWDSNTAV

Supplementary Table 2. Ig variable gene usage for anti-SD1 mAbs.

		Late breakthrough
**Participants**		
Female		13
Male		5
**Median age (Y)**		43 (Range 20-66)
**Vaccine History**		
First dose		
	Pfizer/BioNtech	15
	Oxford/AstraZeneca	3
Second dose		
	Pfizer/BioNtech	15
	Oxford/AstraZeneca	3
Third dose		
	Pfizer/BioNtech	13
	Moderna	4
Fourth dose		
	Pfizer/BioNtech (bivalent)	5
	Moderna (bivalent)	1

Supplementary Table 3. Information on polyclonal serum samples used in SD1 depletion assays.

IC50 ± SEM (µg/mL)													
**mAb**	**XBB.1.5**	**XBB.1.5 +T323I**	**XBB.1.5 +K529N**	**XBB.1.5 +T547I**	**XBB.1.5 +T547K**	**XBB.1.5 +E554K**	**XBB.1.5 +N556K**	**XBB.1.5 +L560Q**	**XBB.1.5 +A570D**	**XBB.1.5 +A570V**	**XBB.1.5 +T572I**	**XBB.1.5 +T573I**	**XBB.1.5 +E583D**
**SD1-1**	0.045 ± 0.022	0.045 ± 0.003	0.051 ± 0.001	0.017 ± 0.001	0.025 ± 0.002	>10	0.031 ± 0.006	0.156 ± 0.011	0.016 ± 0.003	0.035 ± 0.000	0.050 ± 0.002	0.028 ± 0.005	0.033 ± 0.007
**SD1-2**	0.043 ± 0.005	0.043 ± 0.001	0.046 ± 0.000	0.014 ± 0.004	0.074 ± 0.009	>10	0.029 ± 0.001	0.116 ± 0.005	0.016 ± 0.005	0.033 ± 0.002	0.048 ± 0.002	0.034 ± 0.005	0.030 ± 0.005
**SD1-3**	0.096 ± 0.012	0.065 ± 0.005	0.163 ± 0.003	0.038 ± 0.005	0.056 ± 0.006	>10	0.090 ± 0.014	0.406 ± 0.051	0.037 ± 0.007	0.074 ± 0.007	0.056 ± 0.006	0.051 ± 0.001	0.045 ± 0.009

Supplementary Table 4. IC50 values for neutralization assays shown in Fig. 6c.

Supplemental Materials References

1. Hong, Q. *et al.* Molecular basis of receptor binding and antibody neutralization of Omicron. *Nature* **604**, 546-552 (2022).

2. Seow, J. *et al.* A neutralizing epitope on the SD1 domain of SARS-CoV-2 spike targeted following infection and vaccination. *Cell Rep* **40**, 111276 (2022).

3. Bianchini, F. *et al.* Human neutralizing antibodies to cold linear epitopes and subdomain 1 of the SARS-CoV-2 spike glycoprotein. *Sci Immunol* **8**, eade0958 (2023).
