# Supplementary material for: The SARS-CoV-2 neutralizing antibody response to SD1 and its evasion by BA.2.86
Source: Nat Commun. 2024 Mar 28;15:2734. doi: 10.1038/s41467-024-46982-6 (PMC10978878; doi:10.1038/s41467-024-46982-6)
Supplement: Supplementary file 3 — Reporting Summary [file 41467_2024_46982_MOESM3_ESM.pdf]

nature portfolio

Corresponding author(s): David Stuart

Last updated by author(s): 14 Feb 2024

Reporting Summary

Nature Portfolio wishes to improve the reproducibility of the work that we publish. This form provides structure for consistency and transparency in reporting. For further information on Nature Portfolio policies, see our Editorial Policies and the Editorial Policy Checklist.

Statistics

For all statistical analyses, confirm that the following items are present in the figure legend, table legend, main text, or Methods section.

n/a	Confirmed	
☐	☒	The exact sample size (*n*) for each experimental group/condition, given as a discrete number and unit of measurement
☒	☐	A statement on whether measurements were taken from distinct samples or whether the same sample was measured repeatedly
☐	☒	The statistical test(s) used AND whether they are one- or two-sided<br>*Only common tests should be described solely by name; describe more complex techniques in the Methods section.*
☒	☐	A description of all covariates tested
☒	☐	A description of any assumptions or corrections, such as tests of normality and adjustment for multiple comparisons
☐	☒	A full description of the statistical parameters including central tendency (e.g. means) or other basic estimates (e.g. regression coefficient) AND variation (e.g. standard deviation) or associated estimates of uncertainty (e.g. confidence intervals)
☐	☒	For null hypothesis testing, the test statistic (e.g. *F*, *t*, *r*) with confidence intervals, effect sizes, degrees of freedom and *P* value noted<br>*Give P values as exact values whenever suitable.*
☒	☐	For Bayesian analysis, information on the choice of priors and Markov chain Monte Carlo settings
☒	☐	For hierarchical and complex designs, identification of the appropriate level for tests and full reporting of outcomes
☒	☐	Estimates of effect sizes (e.g. Cohen's *d*, Pearson's *r*), indicating how they were calculated

Our web collection on statistics for biologists contains articles on many of the points above.

Software and code

Policy information about availability of computer code

Data collection	cryoEM: EPU<br>FRNT: Elispot
Data analysis	EM: Cryosparc<br>FRNT: Viridot, SPSS, GraphPad Prism

For manuscripts utilizing custom algorithms or software that are central to the research but not yet described in published literature, software must be made available to editors and reviewers. We strongly encourage code deposition in a community repository (e.g. GitHub). See the Nature Portfolio guidelines for submitting code & software for further information.

Data

Policy information about availability of data

All manuscripts must include a data availability statement. This statement should provide the following information, where applicable:

- Accession codes, unique identifiers, or web links for publicly available datasets
- A description of any restrictions on data availability
- For clinical datasets or third party data, please ensure that the statement adheres to our policy

The data that support this study are available from the corresponding author upon request. The atomic models and cryo-EM density maps have been deposited into

the Protein Data Bank (PDB) and Electron Microscopy Data Bank (EMDB) as follows: BA.4-spike/SD1-1: PDB 8CIN and EMD-16680, BA.2.12.1-spike/SD1-2: PDB 8R1C and EMD-18807, BA.2.12.1-spike/SD1-3: PDB 8R1D and EMD-18808. Previously published: BA.2-07 Fab in complex with SARS-COV-2 BA,2,12,1 spike glycoprotein: PDB 8CIM [https://doi.org/10.2210/pdb8CIM/pdb]. Source data are provided with this paper.

Research involving human participants, their data, or biological material

Policy information about studies with human participants or human data. See also policy information about sex, gender (identity/presentation), and sexual orientation and race, ethnicity and racism.

Reporting on sex and gender	Sex and gender were not considered in the study design. Sex and gender of participants were determined based on self-report.
Reporting on race, ethnicity, or other socially relevant groupings	These were not considered in the study design
Population characteristics	Not considered in study design
Recruitment	Following informed consent, individuals with omicron BA.4, BA.5, BA.2.73, BA.5.1, BA.5.2, XBB.1.5, BE.1, CH.1.1, CH.1.1.2 and BQ.1.1 were co-enrolled into one or more of the following three studies: the ISARIC/WHO Clinical Characterisation Protocol for Severe Emerging Infections [Oxford REC C, reference 13/SC/0149], the "Innate and adaptive immunity against SARS-CoV-2 in healthcare worker family and household members" protocol (approved by the University of Oxford Central University Research Ethics Committee), or the Gastro-intestinal illness in Oxford: COVID sub study [Sheffield REC, reference: 16/YH/0247].
Ethics oversight	Our research complies with all relevant ethical regulations. The study protocol was approved by by the University of Oxford Central University Research Ethics Committee.

Note that full information on the approval of the study protocol must also be provided in the manuscript.

Field-specific reporting

Please select the one below that is the best fit for your research. If you are not sure, read the appropriate sections before making your selection.

[x] Life sciences   [ ] Behavioural & social sciences   [ ] Ecological, evolutionary & environmental sciences

For a reference copy of the document with all sections, see nature.com/documents/nr-reporting-summary-flat.pdf

Life sciences study design

All studies must disclose on these points even when the disclosure is negative.

Sample size	No statistical method was used to predetermine sample size.
Data exclusions	No data were excluded
Replication	Where appropriate independent experiments were duplicated.
Randomization	The experiments were not randomized.
Blinding	The Investigators were not blinded to allocation during experiments and outcome assessment.

Reporting for specific materials, systems and methods

We require information from authors about some types of materials, experimental systems and methods used in many studies. Here, indicate whether each material, system or method listed is relevant to your study. If you are not sure if a list item applies to your research, read the appropriate section before selecting a response.

Materials & experimental systems

n/a	Involved in the study	
[ ]	[x]	Antibodies
[ ]	[x]	Eukaryotic cell lines
[x]	[ ]	Palaeontology and archaeology
[x]	[ ]	Animals and other organisms
[x]	[ ]	Clinical data
[x]	[ ]	Dual use research of concern
[x]	[ ]	Plants

Methods

n/a	Involved in the study	
[x]	[ ]	ChIP-seq
[ ]	[x]	Flow cytometry
[x]	[ ]	MRI-based neuroimaging

Antibodies

Antibodies used	Antibodies were produced ourselves, as described, with the exception of: IgM-FITC from BD, #555782, IgA-FITC from Dako, #F0188, IgD-FITC from Dako, #F0189, IgG-BV786 from BD, #564230 and Strep-MAB-DY549 from iba, #2-1566-050, strepMAB-Classic (2-1507-001, IBA), Goat anti-mouse IgG-AP (A9316, Sigma-Aldrich), anti-human IgG-AP (A9544, Sigma-Aldrich).
Validation	As described by manufacturer.

Eukaryotic cell lines

Policy information about cell lines and Sex and Gender in Research

Cell line source(s)	HEK293 cells, source ATCC, Cat#CRL-3216; HEK293T/17 cells, source ATCC, Cat#CRL-11268™; Expi293F, source Gibco, Cat#A14527; Vero cells, source ATCC, Cat#CCL81; VeroE6/TMPRSS2, source NIBSC, Cat#100978
Authentication	Not authenticated
Mycoplasma contamination	Not tested
Commonly misidentified lines (See ICLAC register)	No commonly misidentified cell lines in this study.

Flow Cytometry

Plots

Confirm that:

- [x] The axis labels state the marker and fluorochrome used (e.g. CD4-FITC).
- [x] The axis scales are clearly visible. Include numbers along axes only for bottom left plot of group (a 'group' is an analysis of identical markers).
- [x] All plots are contour plots with outliers or pseudocolor plots.
- [ ] A numerical value for number of cells or percentage (with statistics) is provided.

Methodology

Sample preparation	PBMC were isolated from PCR-confirmed, BA.4/5 infected convalescent blood samples. PBMC were stained with LIVE/DEAD Fixable Aqua dye (Invitrogen) followed by recombinant trimeric S-twin-Strep of BA.4/5. Cells were then incubated with CD3-FITC, CD14-FITC, CD16-FITC, CD56-FITC, IgM-FITC, IgA-FITC, IgD-FITC, IgG-BV786 and CD19-BUV395, along with Strep-MAB-DY549 to stain the twin strep tag of the S protein.
Instrument	BD FACSAria Fusion sorter
Software	DIVA
Cell population abundance	100% sorted cells were relevant cell population. As single cell sorting was used in this study, the purify of the samples was not tested by FACS, but PCR process following sorting confirmed the purity of the population.
Gating strategy	The preliminary FSC/SSC gates of the staining population was identified by the general location of lymphocytes as shown in literatures; IgG+ memory B cells were gated as CD19+, IgG+, CD3-, CD14-, CD56-, CD16-, IgM-, IgA- and IgD-, and S+ was further selected; the boundaries between 'positive' and 'negative' staining cell populations were defined by negative control samples.

- [x] Tick this box to confirm that a figure exemplifying the gating strategy is provided in the Supplementary Information.
