# Supplementary material for: The SARS-CoV-2 neutralizing antibody response to SD1 and its evasion by BA.2.86
Source: Nat Commun. 2024 Mar 28;15:2734. doi: 10.1038/s41467-024-46982-6 (PMC10978878; doi:10.1038/s41467-024-46982-6)
Supplement: Supplementary file 2 — Peer Review File [file 41467_2024_46982_MOESM2_ESM.pdf]

nature portfolio

Peer Review File

The SARS-CoV-2 neutralizing antibody response to SD1 and its evasion by BA.2.86

Open Access This file is licensed under a Creative Commons Attribution 4.0 International License, which permits use, sharing, adaptation, distribution and reproduction in any medium or format, as long as you give appropriate credit to the original author(s) and the source, provide a link to the Creative Commons license, and indicate if changes were made. In the cases where the authors are anonymous, such as is the case for the reports of anonymous peer reviewers, author attribution should be to 'Anonymous Referee' followed by a clear attribution to the source work. The images or other third party material in this file are included in the article's Creative Commons license, unless indicated otherwise in a credit line to the material. If material is not included in the article's Creative Commons license and your intended use is not permitted by statutory regulation or exceeds the permitted use, you will need to obtain permission directly from the copyright holder. To view a copy of this license, visit http://creativecommons.org/licenses/by/4.0/.

Editorial Note: This manuscript has been previously reviewed at another journal that is not operating a transparent peer review scheme. This document only contains reviewer comments and rebuttal letters for versions considered at *Nature Communications*.

REVIEWERS' COMMENTS

Reviewer #1 (Remarks to the Author):

The authors has improved the manuscript in this revision submitted to Nature Communications. They have addressed my concerns for their initial submission. I have no further comments.

Reviewer #2 (Remarks to the Author):

In the revised manuscript, the authors have addressed most of my concerns that were proposed during the previous review processes in Nature Immunology. I believe the manuscript is generally suitable for publication in Nature Communications in its current form.
